# Dual and Multi-Emission Hybrid Micelles Realized through Coordination-Driven Self-Assembly

**DOI:** 10.3390/ma13020440

**Published:** 2020-01-16

**Authors:** Youxiong Zheng, Yan Tang, Jianwei Yu, Lan Xie, Huiyou Dong, Rongsheng Deng, Fuhua Jia, Bingxin Liu, Li Gao, Junyuan Duan

**Affiliations:** 1 Qinghai Provincial Key Laboratory of New Light Alloys, Qinghai Provincial Engineering Research Center of High Performance Light Metal Alloys and Forming, Qinghai University, Xining 810016, China; youxiongzheng@126.com (Y.Z.); a1172050909@163.com (Y.T.); yjw13897471771@163.com (J.Y.); 18990052263@163.com (L.X.); zblzc10@163.com (H.D.); ydxrdsr@126.com (R.D.); hellofuhua@126.com (F.J.); 2 State Key Laboratory of Material Processing and Die & Mould Technology, School of Materials Science and Engineering, Huazhong University of Science and Technology, Wuhan 430074, China

**Keywords:** 8-hydroxyquinoline, quantum dots, hybrid micelles, dual-emission, multi-emission

## Abstract

Building novel functional nanomaterials with a polymer is one of the most dynamic research fields at present. Here, three amphiphilic block copolymers of 8-hydroxyquinoline derivative motifs (MQ) with excellent coordination function were synthesized by Reversible Addition-Fragmentation Chain Transfer Polymerization (RAFT) polymerization. The coordination micelles were prepared through the self-assembly process, which the MQ motifs were dispersed in the hydrophobic polystyrene (PSt) blocks and hydrophilic Poly(N-isopropylacrylamide (PNIPAM)) blocks, respectively. The dual-emission micelles including the intrinsic red light emission of quantum dots (QDs) and the coordination green light emission of Zn^2+^-MQ complexes were built by introducing the CdSe/ZnS and CdTe/ZnS QDs in the core and shell precisely in the coordination micelles through the coordination-driven self-assembly process. Furthermore, based on the principle of three primary colors that produce white light emission, vinyl carbazole units (Polyvinyl Carbazole, PVK) with blue light emission were introduced into the hydrophilic PNIPAM blocks to construct the white light micelles that possess special multi-emission properties in which the intrinsic red light emission of QDs, the coordination green light of Zn^2+^-MQ complexes, and the blue light emission of PVK were synergized. The dual and multi-emission hybrid micelles have great application prospects in ratiometric fluorescent probes and biomarkers.

## 1. Introduction

Self-assembled nanostructured materials built from block polymers have attracted much attention. Block copolymers can self-assemble into ordered assemblies of various shapes, such as micelles and vesicles, according to the properties and proportions of their components [1,2,3]. The development of new functional nanomaterials based on the principle of self-assembly have shown a bright prospect in the field of high technology [4,5,6,7,8]. Polymeric micelles equipped with responsive elements are similar to multi-functional platforms, and their applications gradually extend from drug carriers to environmental responses (such as light, electricity, magnetism, heat, salt, and pH responsive environmentally sensitive smart materials), biological detection, imaging, and other fields [4,5,6,7,8] to achieve multi-functional integration. For example, poly(N,N-dimethylacrylamide)-blockpolystyrene-block-poly[N-(4-vinylbenzyl)-N,N-dibutylamine] (PDMA-b-PS-b-PVBA) triblock copolymer prepared by Zhang [9] were self-assembled into micelles with pH responsiveness. The PDMA-b-PS-b-PVBA aggregates prepared by this research group showed morphological changes of vesicles to micelles with increasing temperature (higher than the critical phase transition temperature) [10]. Yuan’s research group [11] used the β-cyclodextrin functionalized Poly(ethylene glycol)-block-Poly(glycidyl methacrylate) (PEG-b-PGMA) block copolymer and ferrocene-terminated Polycaprolactone (PCL) to self-assemble micelles through the host and guest recognition interaction, which had electrochemical redox responsiveness.

Using block copolymer as a template and inducing inorganic nanoparticles into polymer through its self-assembly process has become one of the important methods to obtain nanoparticle/polymer composites [12]. The resulting composite materials, (1) on the structure, on the one hand, assemblies with abundant morphologies can be obtained [13]. For example, Tan et al. [14] successfully introduced Ag nanoparticles into poly (acrylic acid)-block-polystyrene (PAA-b-PSt) and poly (acrylic acid)-block-poly (acrylic acid-ran-styrene) PAA-b-P(AA-r-St) diblock copolymers to obtain Ag/polymer composites with various forms, such as spherical micelles, worms, vesicles, and lacunal nanospheres. On the other hand, the position of inorganic nanoparticles in the assemblies can be precisely controlled, and the electron or energy donor can be separated from the acceptor, so as to achieve the purpose of adjusting the optical properties. For example, Farinha et al. [15] introduced quantum dots into the core of the micelle, and Au nanoparticles were placed in the shell. When the distance between them was adjusted to about 20 nm, the quantum dot fluorescence was not only not quenched by the Au nanoparticles but increased by eight times. (2) In nature, it is undoubtedly an effective combination of the advantages of the two. Not only the dispersion and compatibility of nanoparticles can be improved to raise the surface activity of particles, but also collected the excellent properties of different components to achieve the functional integration between nanoparticles and polymers through this synergistic effect. (3) In application, micellar-coated nanoparticles extend to biomarkers, nanodevices, catalysis, chemical sensing, and other fields. Zhou et al. [16] encapsulated Ag into poly((2-dimethylamino) ethyl methacrylate)-co-poly(hexafluorobutyl acrylate) (P(DMAEMA-co-HFBA)) to form Ag/P(DMAEMA-co-HFBA) amphiphilic random copolymer hybrid micelles, which showed excellent surface activity and pH sensitivity. Pereira et al. [17] distributed Au nanoparticles to the core of the micelle and regulated the concentration of the macro-RAFT reagent to adjust the length of the polymer chain growing around the Au core, which was of major interest for the design of biosensors, such as pH, temperature, and photoluminescence quenching.

As far as the composite method is concerned, there are currently two main methods for compounding nanoparticles into micelles. One is an in situ method in which nanoparticles are generated in situ in micelles [18]. Zigon et al. [19] introduced Zn^2+^ into poly (methyl methacrylate)-block-poly (methyl methacrylate-co-(zinc methacrylate acetate)) (PMMA-b-P(MMA-co-ZnMAAc)) micelles formed by self-assembly to obtain ZnO/PMMA rod micelles through in situ reaction. Mai [20] introduced HAuCl_4_ into polystyrene-block-poly (acrylic acid) (PS-b-PAA) block copolymer and obtained the micelles of Au nanoparticles in the core by in situ. The other is to introduce nanoparticles into the micelles by means of “co-assembly” [21]. For example, Taton et al. [22] dispersed PS-b-PAA diblock copolymers and Au nanoparticles coated with sodium citrate into a N,N-Dimethylformamide (DMF) solution, which the polymer hydrophobic blocks and Au nanoparticles self-assemble into the micellar core in selective solvent water, while the PAA blocks always dissolved in water and maintained micellar stability. In addition to the above two methods, Zhu et al. [23] used supramolecular assembly technology to introduce Au nanoparticles into the club-like or vermicular micelles of Polystyrene-b-polyvinylpyridine (PS-P4VP). Other methods such as the electrostatic interaction method and layer upon layer self-assembly method have also been reported [24]. Zhang et al. [25] constructed white-light emitting dye micelles in aqueous solution by loading bispolyimide (PBI) onto biscarbazole compounds. Wang et al. [26,27] achieved white light emission through a single layer of diblock copolymer micelles containing green- and red-light-emitting dyes in the separate micellar cores and blue-light-emitting polymer around their periphery. Additionally, the white light emission was enhanced through loading green- and red-light-emitting donor–acceptor pairs in the separate micellar cores and blue-light-emitting polymers around their periphery.

**Scheme 1:**
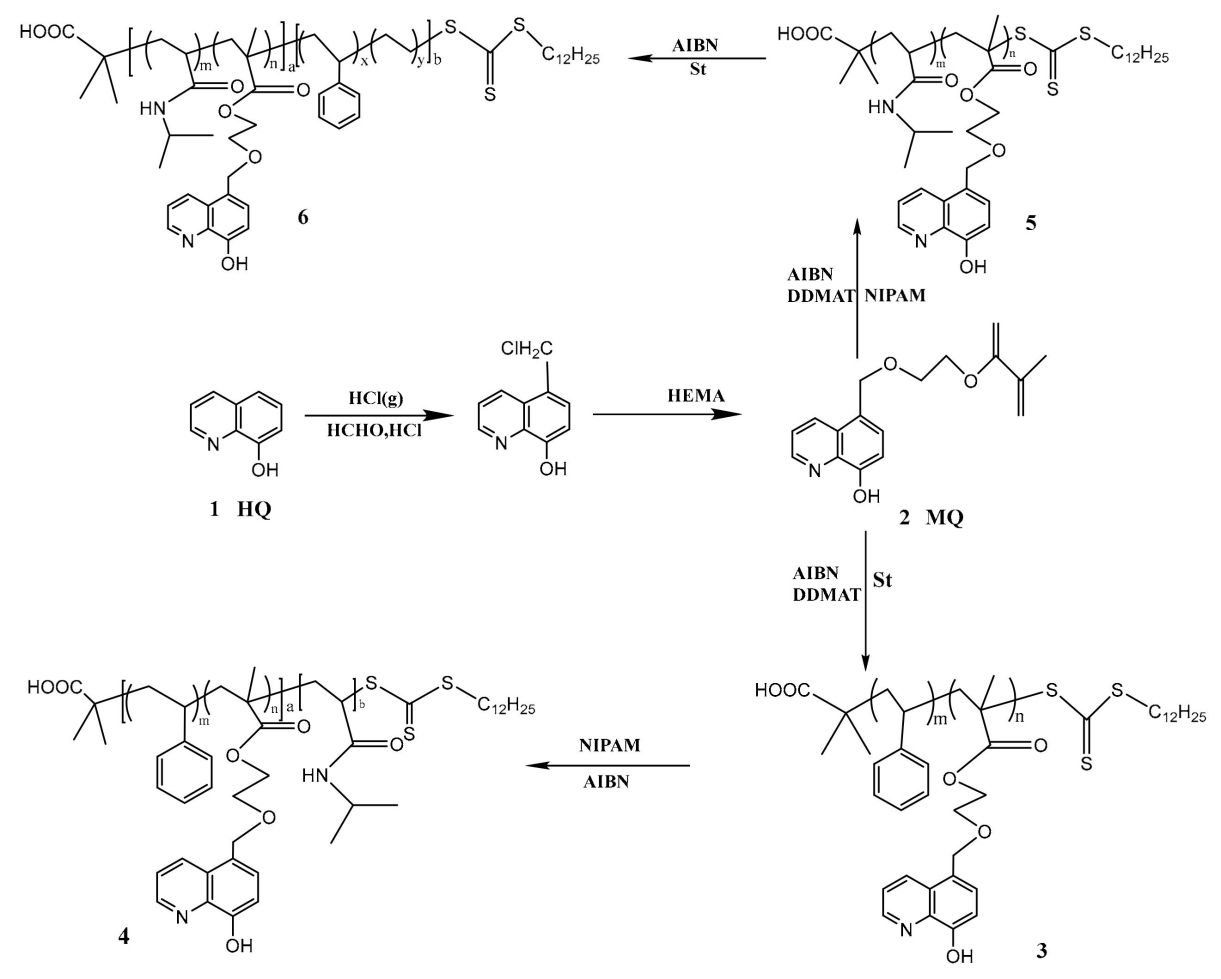
Synthesis diagram of P(MQ-co-St)-b-PNIPAM (Polymer 4) and P(MQ-co-NIPAM)-b-PS (Polymer 6).

**Scheme 2:**
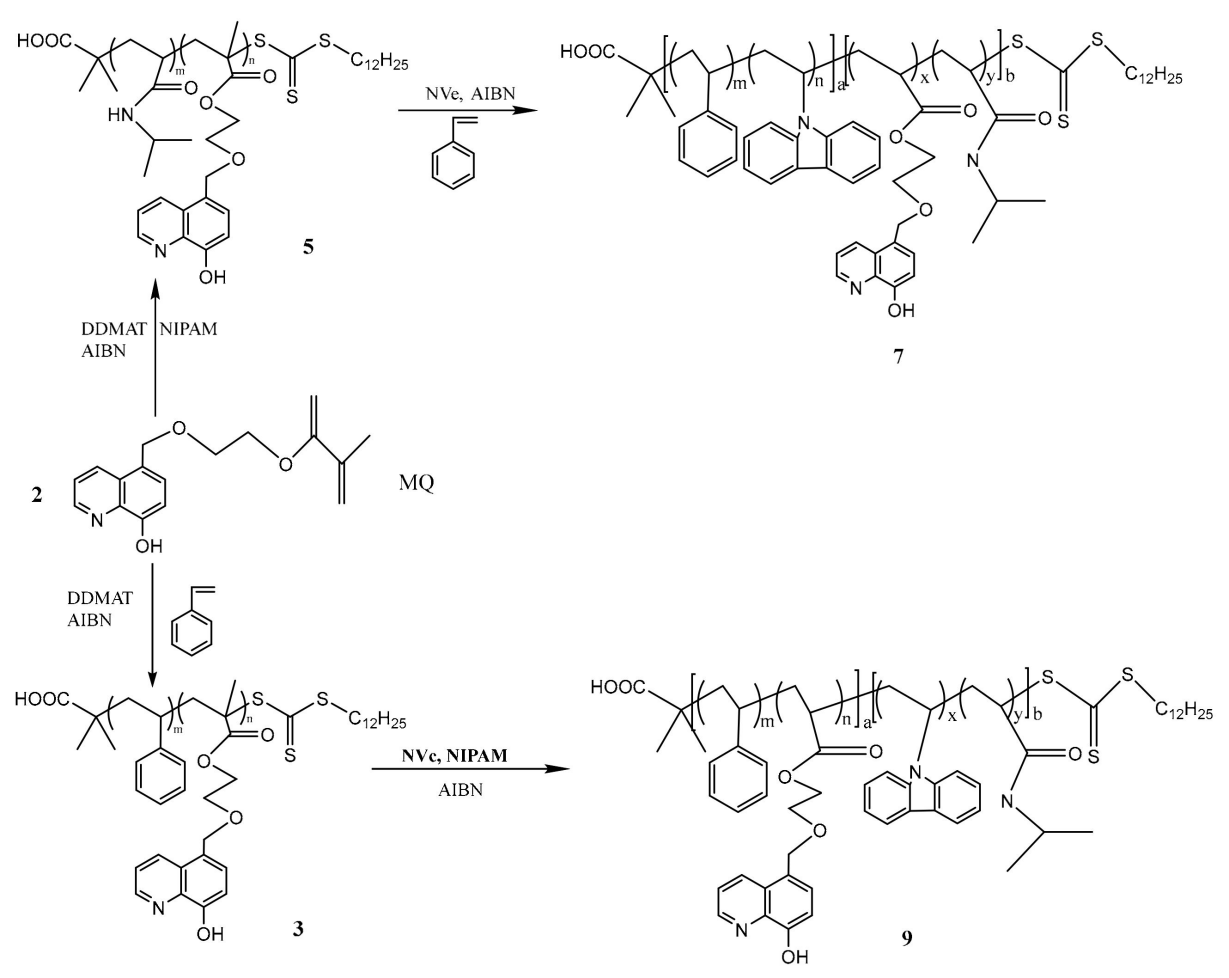
Synthesis diagram of P(PVK-co-St)-b-P(MQ-co-NIPAM) (Polymer 7) and P(MQ-co-St)-b-P(NIPAM-PVK) (Polymer 9).

**Scheme 3:**
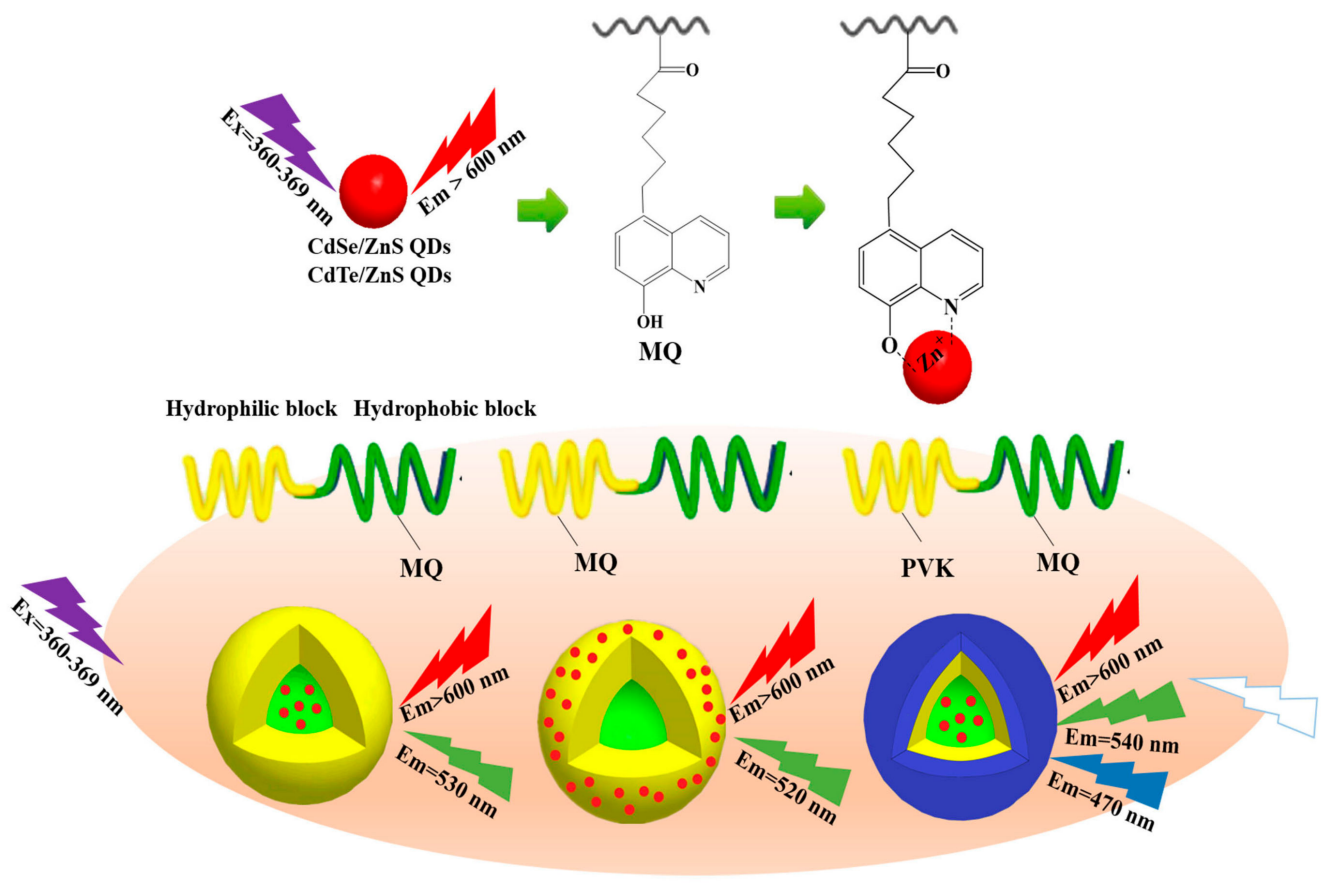
Synthesis diagram of dual-emission and white light emission micelle.

However, in the current research, the fluorescent nanoparticle/block copolymer hybrid aggregates are mostly single emission, and the luminescence is not adjustable, and there is not much studies on white light emission micelles. Secondly, the stability of hybrid micelles is also facing the test of complex application environments. Nanoparticles are mostly introduced into micelles through interactions such as hydrophilic–hydrophobic interactions and hydrogen bonding. Coordination-driven self-assembly to form nanoparticle/block copolymer hybrid aggregates is expected to be important in micelle formation and stability. Therefore, in this work, we synthesized three types of amphiphilic block copolymers (amphiphilic macromolecular ligands) containing 8-hydroxyquinoline derivatives units (MQ) with excellent coordination function using reversible addition-fragmentation chain transfer polymerization (RAFT) (Scheme 1 and Scheme 2). We successfully prepared the coordination micelles M4 and M6 using the coordination-driven self-assembly effect in which the MQ motifs were dispersed in the hydrophobic PSt blocks and the hydrophilic PNIPAM blocks, respectively (Scheme 1 and Scheme 3). The CdSe/ZnS and CdTe/ZnS QDs were precisely located in the core and shell of the coordination micelles M4 and M6 through the coordination-driven self-assembly process to build the dual-emission micelles in which the intrinsic red light emission of QDs was synergized with the coordination of green light emission of Zn^2+^-MQ complexes. Moreover, based on the principle of three primary colors that produce white light emission, we introduced the vinyl carbazole units (PVK) with blue light emission into the hydrophilic PNIPAM blocks to construct the white light micelles that possess special multi-emission properties in which the intrinsic red light emission of QDs, the coordination green light of Zn^2+^-MQ complexes, and the blue light emission of PVK were synergized (Scheme 2 and Scheme 3). The dual and multi-emission hybrid micelles have great application prospects in ratiometric fluorescent probes and biomarkers.

## 2. Materials and Methods

### 2.1. Materials and Characterization

The reagents used in the experiment are as follows. Briefly, cadmium chloride (CdCl_2_·2.5H_2_O, Analytical Reagent (AR), Shanghai Reagent Factory, Shanghai, China), powder (Te, AR, Sinopharm Chemical Reagent Co.,Ltd, Shanghai, China), zinc chloride (ZnCl_2_, AR, Shanghai Reagent Factory, Shanghai, China), sodium borohydride (NaBH_4,_ AR, Sinopharm Chemical Reagent Co.,Ltd), 1-octadecene (ODE, AR, Sigma-Aldrich, St. Louis, MO, USA), octadecylamine (ODA, AR, Acros, NJ, USA), oleic acid (OA, AR, Alfa Aesar, Haverhill, MA, USA), cadmium oxide (CdO, AR, Sigma), selenium powder (Se, AR, Acros), zinc oxide (ZnO, AR, Sigma-Aldrich), sulfur powder (S, AR, Sigma-Aldrich), N-octylphosphine (TOP, AR, Sigma-Aldrich ), zinc acetate (Zn(Ac)_2_, AR, Alfa Aesar, Shanghai, China), Dodecanethiol (AR, Aladdin, Shanghai, China), cetyltrimethylammonium chloride (CTACl, AR, Tianjin Guangfu Fine Chemical Research Institute, Tianjin, China), carbon disulfide (CS_2_, AR, Tianjin Guangfu Fine Chemical Research Institute), azobisisobutyronitrile (AIBN, 99%, Aladdin) was recrystallized from ethanol, styrene (St, AR, Xilong Chemical, Shantou, China) and isopropyl acrylamide (NIPAM, AR, Sinopharm Chemical Reagent Co.,Ltd) was recrystallized from n-hexane before use, as well as vinyl carbazole (NVc, AR, Sinopharm Chemical Reagent Co.,Ltd) and 8-hydroxyquinoline (HQ, AR, Sinopharm Chemical Reagent Co.,Ltd).

Fourier transform infrared spectroscopy (FT-IR) was analyzed by a Magna 560 infrared spectrometer (Nicolet Ltd, GreenBay, WI, USA). X-ray diffraction (XRD) was analyzed through Siemens D-5005 X-ray diffractometer CuK α (λ = 1.5418 Å, Bruker Ltd, Bremen, Germany). ^1^H-NMR was tested by AVANCE Bruker (500 MHz, Bruker Ltd, Bremen, Germany) in a CDCl_3_ solution. Ultravioletvisible (UV–Vis) absorption spectrum was tested by a SHIMADZU UV-2550 UV–Vis spectrometer (Shimadzu, Japan) in the range of 200–800 nm. Fluorescence spectra was analyzed by FLSP920 Edinburgh fluorescence spectrometer (Edinburgh Instruments Ltd, Corston, UK). The molecular weight of the macromolecular ligand was tested by waters gel chromatography (GPC, Waters Corporation, Milford, MA, USA) at 40 °C with THF as the eluent and polystyrene as the reference, the flow rate was controlled at 1.0 mL·min^−1^. The nanoparticle morphology was characterized by a JEM-2100F transmission electron microscope (JEOL Ltd, Tokyo, Japan).

### 2.2. Synthesis of QDs

The preparation of CdSe/ZnS QDs capped with oleic acid and water phase CdTe/ZnS QDs were prepared by reference to previous methods [28,29] (see Supplementary Materials).

### 2.3. Synthesis of RAFT Reagent trithiocarbonate S-1-dodecyl-S’-(α,α’-dimethyl-α”-acetic acid) (DDMAT)

RAFT reagent DDMAT was prepared by reference to previous methods [30] (see Supplementary Materials). Melting point (Mp): 64 °C. ^1^H NMR (500 MHz, CDCl_3_, δ, ppm): 0.99 (t, 3 H), 1.37 1.47 (m, 20 H), 1.75 (s, 6 H), 3.42 (t, 2 H).

### 2.4. Synthesis of Amphiphilic Macromolecular Ligands

#### 2.4.1. Synthesis of P(MQ-co-St)-b-PNIPAM

As shown in Scheme 1, using MQ and St as monomers, AIBN as an initiator, DDMAT as a RAFT reagent, the ratio of monomer, DDMAT, and AIBN was controlled at 1000:100:1 in a 25 mL THF solution [31]. After three freeze-thaw cycles, the mixture was degassed with N_2_ and reacted at 70 °C for 20 h. After completion of the reaction, the reaction solution was added dropwise to a large amount of methanol to precipitate, and the obtained precipitate was washed by THF. After repeating this procedure five time, the macromolecular RAFT reagent P(MQ-co-St)-RAFT (Polymer 3) dried under vacuum was obtained. Taking NIPAM as monomer, P(MQ-co-St)-RAFT as macromolecular RAFT reagent, and AIBN as initiator, the ratio between the above three was controlled at 1000:100:1 in a 25 mL THF solution, and the content of P(MQ-co-St)-RAFT was controlled at 10%, 20%, 30%, 40% (i.e., 4-1, 4-2, 4-3 and 4-4, respectively). After three freeze-thaw cycles, the mixture was degassed with N_2_ and reacted at 70 °C for 20 h. After completion of the reaction, the reaction solution was added dropwise to a large amount of petroleum ether to precipitate, and the obtained precipitate was washed by THF. After repeating this procedure five times, the macromolecular ligands P(MQ-co-St)-b-PNIPAM (Polymer 4) dried under vacuum was obtained [32,33].

#### 2.4.2. Synthesis of P(MQ-co-NIPAM)-b-PS

The synthesis method and conditions of P(MQ-co-NIPAM)-b-PS were the same as the 2.4.1 method. First, the macromolecular RAFT reagent P(MQ-co-NIPAM)-RAFT (Polymer 5) were synthesized. P(MQ-co-NIPAM)-b-PS (Polymer 6) (i.e., 6-1, 6-2, 6-3, and 6-4, respectively) were then obtained by grafting the St segments to Polymer 5 [31].

### 2.5. Synthesis of Amphiphilic Blue Light Macromolecular Ligands

As shown in Scheme 2, the macromolecular RAFT reagent P(MQ-co-St)-RAFT (Polymer 3) and P(MQ-co-NIPAM)-RAFT (Polymer 5) that were firstly obtained refer to the procedure of above. Taking NIPAM and NVc as monomer, P(MQ-co-St)-RAFT as macromolecular RAFT reagent, and AIBN as initiator, the ratio between the above three was controlled at 1000:100:1 in a 25 mL THF solution [31]. After three freeze-thaw cycles, the mixture was degassed with N_2_ and reacted at 70 °C for 20 h. After completion of the reaction, the reaction solution was added dropwise to a large amount of petroleum ether to precipitate, and the obtained precipitate was washed by THF. After repeating this procedure five times, the amphiphilic blue light emission macromolecular ligands P(MQ-co-St)-b-P(NIPAM-PVK) (Polymer 9) dried under vacuum was obtained. Similarly, taking St and NVc as monomer, P(MQ-co-NIPAM)-RAFT as macromolecular RAFT reagent, and AIBN as initiator, the amphiphilic blue light emission macromolecular ligands P(PVK-co-St)-b-P(MQ-co-NIPAM) (Polymer 7) was obtained finally.

### 2.6. Preparation of Coordination Micelles

Polymers 4 and 6 in a THF solution (1 mg/mL) were slowly added dropwise with ultrapure water for self-assembly until the solution with blue opalescence was obtained. After stirring for 3 h, a large amount of ultrapure water was added dropwise into the mixture solution to fix the micelle structure. The above solution was transferred into a dialysis bag after stirring for 3 h again and dialyzed against deionized water for a certain period of time to remove THF from the solution. The finally obtained aqueous micelle solution was brought to the same concentration. The multifunctional coordinating micelles M4 and M6 of Polymers 4 and 6 were thus obtained.

### 2.7. Preparation of Dual-Emission Micelles

CdSe/ZnS@M4: Polymer 4-1 and CdSe/ZnS QDs were uniformly mixed in a THF solution according to the ratio of Polymer 4-1 to QDs mass ratio of 1:0.1, 1:1, 1:3, 1:5, 1:10. Ultrapure water were slowly added into the above mixture solution under vigorous stirring for self-assembly until the solution with blue-opalescence was obtained. After stirring for 3 h, it was dripped into a large amount of ultrapure water under stirring to fix the micelle structure. The solution was transferred to a dialysis bag after stirring for 3 h again and dialyzed against deionized water for a certain period of time to obtain the dual-emission micelles CdSe/ZnS@M4.

M6@CdTe/ZnS: The CdTe/ZnS solution under vigorous stirring was slowly added dropwise to the M6 micelle solution at a certain ratio (1:10, 1:20, 1:30, 1:40, 1:50) to obtain the dual-emission micelles M6@CdTe/ZnS. The fluorescence change of dual-emission micelles was monitored by a photoluminescence (PL) spectrometer.

### 2.8. Preparation of White Light Emission Micelle

Polymer 9 and CdSe/ZnS were uniformly mixed in a THF solution at a mass ratio of 1:0.5, 1:1, 1:3, 1:5, and slowly added with ultrapure water under vigorous stirring for self-assembly until the solution with blue-opalescence was obtained. After stirring for 3 h, it was dropped into a large amount of water under stirring to fix the micelle structure. The solution was transferred into a dialysis bag after stirring for 3 h again and dialyzed against deionized water for a certain time to remove THF from the solution. The white light emission micelles M9 were finally obtained as shown in Scheme 3.

## 3. Results and Discussion

### 3.1. Preparation of CdSe/ZnS and CdTe/ZnS QDs

CdSe QDs with high fluorescence quantum yield were prepared by the reverse injection method (Cd precursor fluid injection into Se precursor fluid) [32,33]. The CdSe produced by the reverse injection method has a much larger yield and is suitable for batch preparation compared with the conventional Cd precursor injection method. Reiss et al. [34,35] prepared CdSe/ZnS QDs by the continuous ion layer adsorption and reaction (SILAR) using CdSe QDs as the core. Figure S1 illustrates the transmission electron microscope (TEM) spectra before and after the coating of ZnS shell by CdSe QDs. The diffraction peaks of the (111), (220), and (311) crystal planes correspond to the ZnS having a cubic sphalerite crystal structure that were observed after the CdSe surface was coated with a ZnS shell of approximately 1.4 nm. Similarly, Figure S3 illustrates the TEM images of CdTe/ZnS QDs. The particle size of CdTe/ZnS QDs is about 3.7 nm. The above analysis indicated that the CdSe/ZnS and CdTe/ZnS QDs have been successfully obtained [36].

### 3.2. Preparation of Amphiphilic Macromolecular Ligands

The RAFT polymerization is a controlled radical polymerization. The RAFT reaction is equipped with an “active” controllable feature due to the reduced molecular weight distribution of the polymer. Therefore, RAFT polymerization is also one of the commonly methods for synthesizing block copolymers with a well-defined structure and a predetermined molecular weight [37]. Here, two types macromolecular ligands with a coordination function were synthesized by RAFT polymerization using DDMAT as the chain transfer agent, MQ with a photoelectric and coordination function, NIPAM with thermosensitivity and styrene as monomer, respectively. The data of the GPC test are listed in Table 1. Figures S4 and S5 are the GPC curves of Polymer 4 and Polymer 6 and their corresponding macro-RAFT agents, respectively. The number and weight average molecular weights (Mn and Mw) are 9200 and 9500 of the macromolecular RAFT reagents P(MQ-co-St)-RAFT and P(MQ-co-NIPAM)-RAFT, respectively. The polydispersity index PDI (Mw/Mn) was 1.032 and 1.012, respectively. The above analysis indicated that the molecular weight distribution of the two polymers were very narrow, which was consistent with the characteristics of RAFT controlled radical polymerization. As shown in Table 1, the different ratio of NIPAM and St monomers were respectively added into P(MQ-co-St)-RAFT and P(MQ-co-NIPAM)-RAFT to carry out the chain extension reaction and the corresponding molecular weights also increased gradually, indicating that the chain extension reaction both can be successfully carried out to obtain a block copolymer by using P(MQ-co-St)-RAFT and P(MQ-co-NIPAM)-RAFT as macromolecular RAFT agents, respectively. The molecular weight distribution of the resulting block copolymer (Polymers 4 and 6) was also relatively narrowed. Where the block copolymer (Polymer 6) prepared by the chain extension reaction using P(MQ-co-NIPAM)-RAFT as macromolecular RAFT reagent and St as monomer has a lower molecular weight distribution.

The structure of the block copolymer obtained by the RAFT polymerization was further confirmed by infrared spectroscopy (see Supporting Information). Figure S6 shows the FT-IR spectrum of P(St-co-MQ)-RAFT and P(St-co-MQ)-b-PNIPAM (Polymer 4). The stretching vibration peak at 3415 cm^−1^ is assigned to the –OH on the MQ motifs in Polymer 3. When it was used as the macromolecular RAFT reagent and chain extension reaction with NIPAM, the –CONH stretching vibration peak at 3313 cm^−1^ is attributed to the NIPAM units, and the absorption strength at 3313 cm^−1^ is enhanced with the increasing content of NIPAM. The above analysis showed that P(St-co-MQ)-b-PS (Polymer 4) has been successfully obtained. Figure S7 is the FT-IR spectrum of P(NIPAM-co-MQ)-RAFT and P(MQ-co-NIPAM)-b-PS (Polymer 6). The band at 3454 cm^−1^ corresponds to the stretching vibration peak of –OH in the MQ motifs in Polymer 5, and the band at 3298 cm^−1^ is assigned to the –CONH stretching vibration peak of NIPAM units. The C–H bending vibration peak at 696 cm^−1^ corresponding to the benzene ring was observed with the chain extension reaction proceeding and becoming more pronounced with the increasing content of the St units. The above analysis indicated that P(NIPAM-co-MQ)-b-PS (Polymer 6) has been successfully obtained.

Figure S8 is the ^1^H NMR spectrum of the macromolecular RAFT reagent DDMAT. The broad chemical shifts of 1 and 4 at 0.8–0.9 and 1.6–1.75 ppm belonged to the protons of –CH_3_ on DDMAT terminal, respectively. The broad chemical shifts of 2 and 3 at 1.2–1.4 and 3.2–3.3 ppm belonged to the protons of –CH_2_ on DDMAT. The above analysis proved that the macromolecular RAFT reagent DDMAT has been successfully synthesized. Figure S9 is the ^1^H NMR spectrum of P(MQ-co-St)-RAFT. The broad chemical shifts of b, d, e, and 5 at 6.0–7.5 ppm are attributed to the protons of quinolone ring on the MQ motifs and the protons of benzene ring on PSt units. The chemical shifts of a, c at 8.7 and 8.5 ppm are ascribed to the protons of the pyridine ring on MQ motifs. The chemical shifts of i at 5–6 ppm are attributed to the active hydrogen (–OH) of quinolone ring on the MQ motifs. The chemical shifts of h, g, and f at 3.5–4.68 ppm are ascribed to the –CH_2_ on the MQ motifs. The chemical shifts of 1, 3, and 4 at less than 2 ppm corresponded to the –CH_2_ and –CH_3_ in P(MQ-co-St)-RAFT main chain. The above analysis proved that St and MQ have been successfully copolymerized. The molar ratio of MQ to St units in the macromolecular RAFT reagent P(MQ-co-St)-RAFT was calculated to be approximately 1:4 by the integrated area and is shown in Table 1. The ^13^C–^1^H correlation spectrum (COSY) of P(MQ-co-St)-RAFT is shown in Figure S10. The chemical shift at 0.9 ppm is attributed to the protons peak of the –CH_3_ at the terminal of the macromolecular RAFT reagent, indicating that the composition of the macromolecular RAFT agent is P(St_49_-co-MQ_12_)-Macro-RAFT. Figures S11–S14 are the ^1^H NMR spectrum of the polymer 4-1, 4-2, 4-3, 4-4, respectively. The chemical shift of 1 corresponded to the hydrogen of the methylidyne on the PNIPAM units observed at 4.0 ppm. The chemical shifts of i at 5–6 ppm corresponded to the active hydrogen (–OH) of quinolone ring on the MQ motifs. The broad chemical shifts of b, d, e, and 2 at 6.0–7.5 ppm are attributed to the protons of quinolone ring on the MQ motifs and the protons of benzene ring on PSt units. The above analysis proved that the extended chain reaction between P(MQ-co-St)-RAFT and NIPAM enabled the successful copolymerization of NIPAM into Polymer 3 to form P(MQ-co-St)-b-PNIPAM (Polymer 4). Similarly, Figure S15 is the ^1^H NMR spectrum of P(MQ-co-NIPAM)-RAFT. The chemical shifts of a, c at 8.5 and 8.7 ppm belonged to the protons of pyridine ring on MQ motifs. The chemical shifts of b, d, and e at 6.0–7.5 ppm are attributed to the protons of the MQ motifs quinoline ring. The chemical shifts of h, g, and f at 3.5–5 ppm are attributed to the protons on the MQ motifs. The chemical shifts of 2 at 4.0 ppm are classified to the protons of –CH on the NIPAM units. The chemical shifts of 1, 3, 4, and 5 at less than 2 ppm corresponded to the –CH_2_ and –CH_3_ in P(MQ-co-NIPAM)-RAFT main chain. The above analysis proved that MQ and NIPAM have been successfully copolymerized to form P(MQ-co-NIPAM)-RAFT. Figures S16–S18 are the ^1^H NMR spectrum of Polymers 6-1, 6-2, and 6-3, respectively. The board chemical shifts of 1, b, d, and e at 6.0–7.5 ppm corresponded to the hydrogen of the benzene ring on the PSt units and the protons of the MQ motifs quinoline ring, respectively. The chemical shifts of 2 at 4.0 ppm belonged to the hydrogen of the methylidyne on the PNIPAM units. The chemical shifts of f at 5.0 ppm corresponded to the protons of –CH_2_ on the MQ motifs. The above analysis also proved that the St units have been successfully copolymerized to P(MQ-co-NIPAM)-RAFT to form P(MQ-co-NIPAM)-b-PS (Polymer 6).

The composition and molecular weight of the amphiphilic block copolymer prepared above were determined by NMR spectroscopy and the data are listed in Table 1, which was not much different from the molecular weight measured by GPC. The characteristic of Polymer 4 is that the MQ motifs with a coordination function are randomly dispersed in the hydrophobic PSt blocks of the block copolymer. The characteristic of Polymer 6 is that MQ motifs are randomly distributed in the hydrophilic PNIPAM blocks of the block copolymer.

### 3.3. Preparation of Coordination Micelles

**Figure 1:**
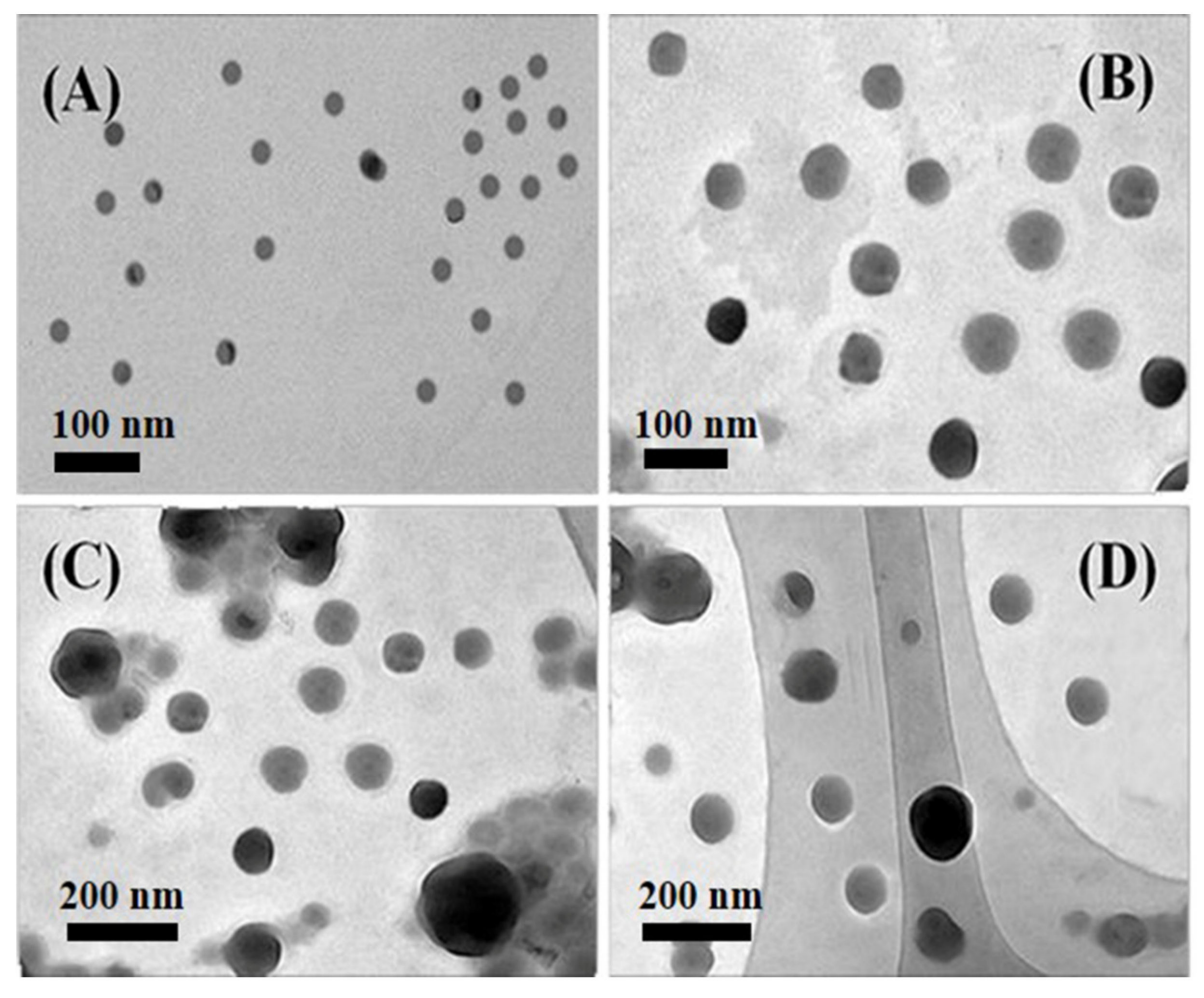
TEM images of different micelles obtained from P(MQ-co-St)-b-PNIPAM assembly: M4-1 (**A**), M4-2 (**B**), M4-3 (**C**), and M4-4 (**D**).

The insoluble blocks in the block copolymer drives the aggregation of the polymer, and the soluble blocks resist the aggregation, so that the self-assembly of the polymer to form micelles is significant in the aggregation behavior of the polymer. There are many factors affecting the self-assembly morphologies of amphiphilic macromolecules, such as temperature, pH, and length of hydrophilic and hydrophobic blocks. Here, THF was chosen as the co-solvent and deionized water was the selective solvent. The self-assembly morphologies of block copolymer in the selective hydrophobic solvent have been studied by adjusting the length ratio of hydrophilic and hydrophobic blocks under the same conditions. Figure 1 shows that the TEM images of self-assemblies were formed in selective solvent water by amphiphilic block copolymers P(MQ-co-St)-b-PNIPAM (Polymer 4) with different hydrophilic and hydrophobic blocks ratio. The amphiphilic block copolymer P(MQ-co-St)-b-PNIPAM are self-assembled in water to form spherical micelles in which the coordination motifs MQ are located in the core. The formation mechanism is that the hydrophobic PSt blocks are gradually aggregated to form the core when deionized water is added dropwise into the amphiphilic block copolymers THF solution, while the hydrophilic blocks are still stretched. Therefore, when the amount of deionized water is added to the critical water content (CWC), the assemblies are formed in which the hydrophobic blocks act as the core and the hydrophilic blocks as the shell. Additionally, the particle size of the micelles M4 were increased as the growing of the hydrophilic PNIPAM blocks under the conditions of the hydrophobic PSt blocks remains unchanged. Among them, the micelles M4-1 formed by Polymer 4-1 have a uniform spherical structure and average particle diameter of about 30 nm. The micelles M4-2, M4-3, and M4-4 formed by Polymers 4-2, 4-3, and 4-4 also possess a spherical structure with particle sizes of 60, 100, and 110 nm, respectively. However, the particle sizes micelles M4-2, M4-3, and M4-4 are not uniform, which may be related to the uneven molecular weight distribution of Polymers 4-2, 4-3, and 4-4. The above analysis suggested that the coordination micelles M4 in which the MQ motifs are located in the core have been successfully prepared.

**Figure 2:**
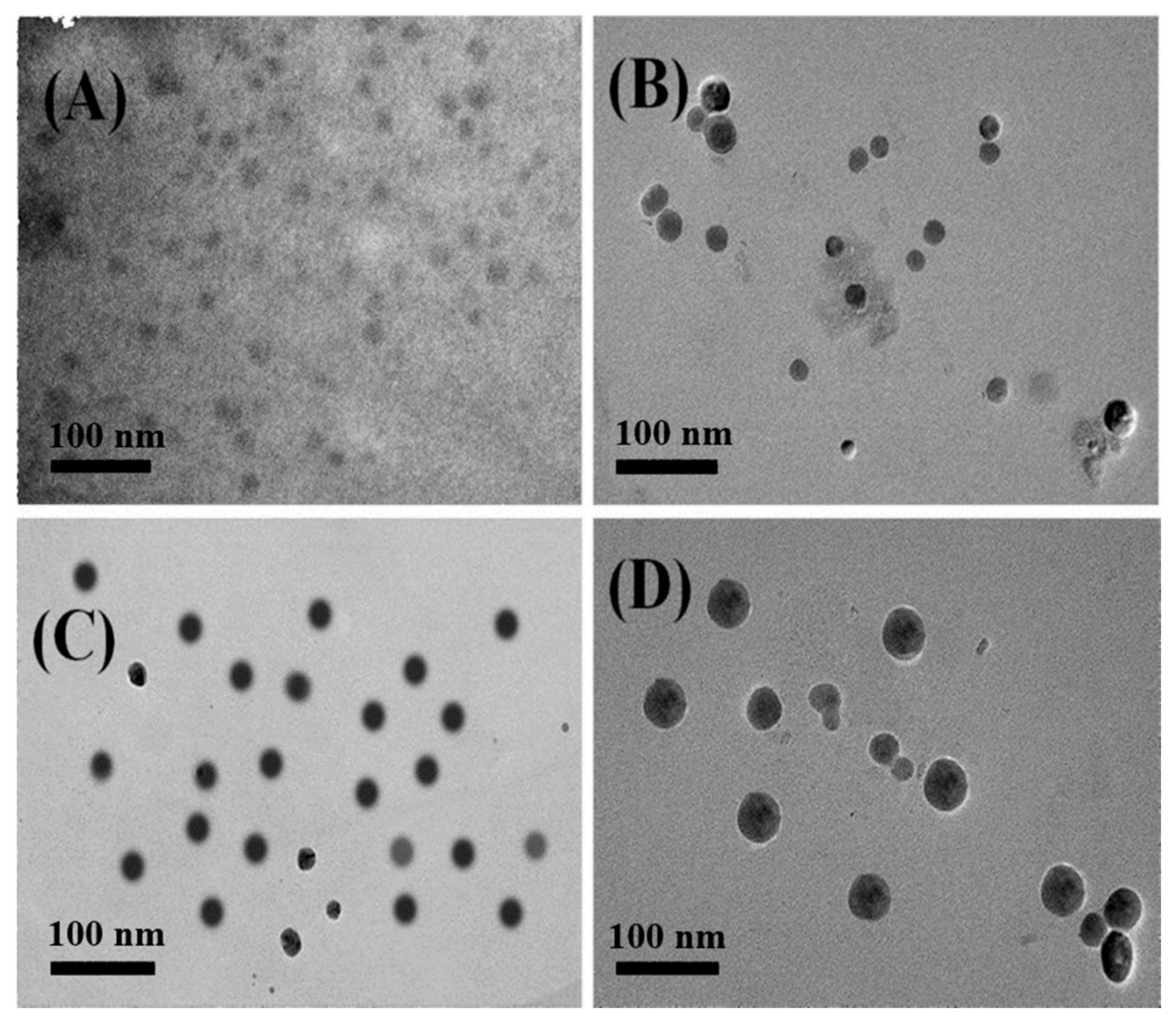
TEM images of different micelles obtained from P(MQ-co-NIPAM)-b-PS assembly: M6-1 (**A**), M6-2 (**B**), M6-3 (**C**), and M6-4 (**D**).

Similarly, Figure 2 shows the TEM images of self-assemblies formed in selective solvent water by amphiphilic block copolymers P(MQ-co-NIPAM)-b-PS (Polymer 6) with different hydrophilic and hydrophobic blocks ratio. The amphiphilic block copolymer P(MQ-co-NIPAM)-b-PS are self-assembled in water to form spherical micelles in which the coordination motifs MQ are located in the shell. The micelles M6-1, M6-2, M6-3, and M6-4 possess spherical structures with particle sizes of 15, 20, 30, and 40 nm that were formed by Polymers 6-1, 6, 6-2, 6-3, and 6-4, respectively. Among them, M6-3 has the most uniform particle size. This showed that the coordination micelles M6 in which the MQ motifs were located in the shell have been successfully prepared and micelles with smaller particle sizes are easily obtained when PSt blocks were only used as a micelle core. The size of the micelles can be controlled by controlling the proportion of hydrophilic and hydrophobic blocks in case that other influencing factors remain unchanged.

### 3.4. Dual-Emission Micelle

The MQ motifs can coordinate with the metal ion on the surface of the nanoparticle to form a stable monolayer complex [38]. The spatial position of nanoparticles in the micelles can be controlled through the ligand position regulation.

#### 3.4.1. CdSe/ZnS@M4

**Figure 3:**
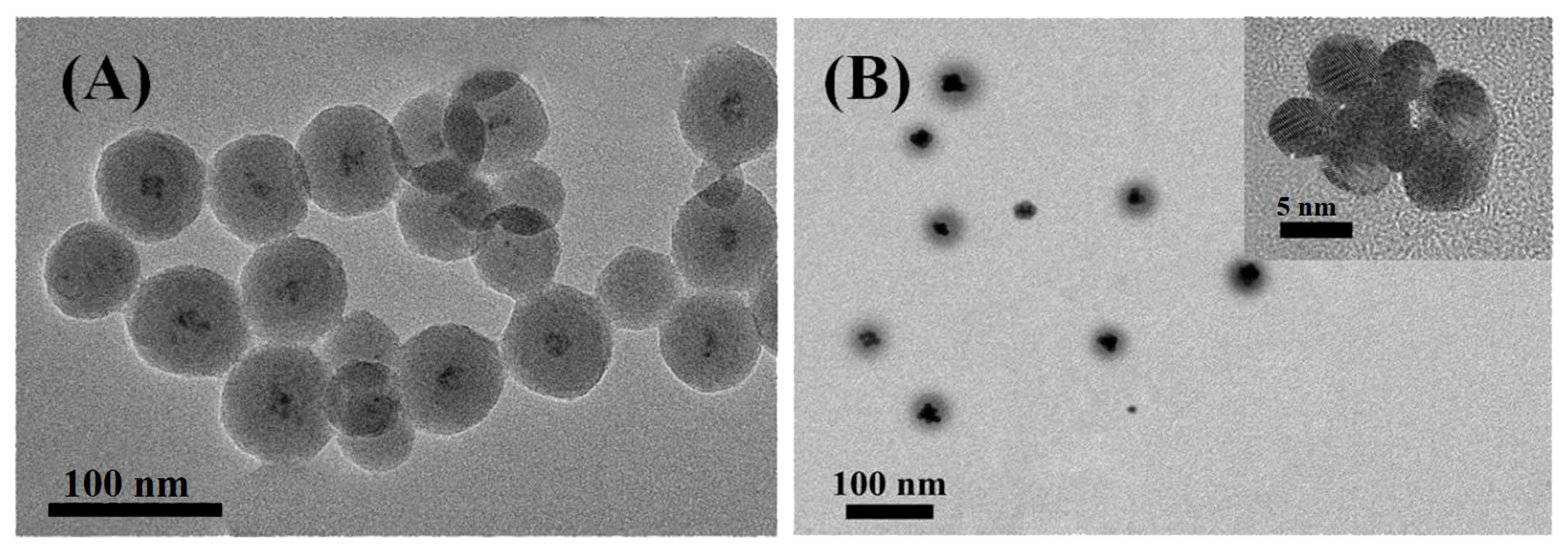
TEM images of CdSe/ZnS@M4-1 with different M4-1/QDs ratio: (**A**) 1: 0.1 and (**B**) 1:10. (insert: HRTEM of single CdSe/ZnS@M4-1, the scale bar refers to 5 nm).

Here, the CdSe/ZnS QDs with capped oleic acid were introduced into the coordination micelles M4 by in situ combination, that was, CdSe/ZnS QDs and Polymer 4-1 (P(St_0.8_-co-MQ_0.2_)_61_-b-PNIPAM-_15_) self-assembled in the selective solvent water by co-precipitation. A series of M4-1@CdSe/ZnS can be obtained by mixing Polymer 4-1 with the CdSe/ZnS QDs at a mass ratio of 1:0.1, 1:1, 1:3, 1:5, and 1:10. Figure 3 shows the TEM images of CdSe/ZnS@M4-1 with different M4-1/QDs ratio of 1: 0.1 and 1:10. When Polymer 4-1 and CdSe/ZnS QDs are mixed in a ratio of 1:0.1 for self-assembly in a selective solvent, the CdSe/ZnS QDs tend to aggregate in the core since it were coordinated with the quinoline ring in Polymer 4-1 which were distributed in the hydrophobic PSt blocks, while the hydrophilic PNIPAM blocks in Polymer 4-1 resisted this aggregation. Thus, the CdSe/ZnS@M4-1 hybrid micelles in which the CdSe/ZnS QDs were located in the core have been successfully formed eventually. Furthermore, the resulting CdSe/ZnS@M4-1 hybrid micelles exhibit the feature of “small core large shell” with an average particle size of 60 nm and a uniform size distribution, which attributes to the high content of Polymer 4-1 and the low content of CdSe/ZnS QDs. Similarly, when M4-1 and CdSe/ZnS QDs are mixed at a ratio of 1:10 for self-assembly, the CdSe/ZnS@M4-1 hybrid micelles possess the feature of “large core and small shell” with the average particle size of nearly 39 nm (Figure 3B), which ascribes a few of Polymer 4-1 that are assigned to the surface of each CdSe/ZnS QDs during self-assembly by the coordination-driven low Polymer 4-1 content. It can be observed from the high-resolution transmission electron microscope (HRTEM) images of a single micelle inserted in Figure 3B that multiple CdSe/ZnS QDs are aggregated in the M4 micellar core by the coordination-driven effect. The above analysis suggested that the CdSe/ZnS QDs have been successfully induced in the self-assembly of the amphiphilic Polymer 4-1 through the coordination-driven effect between CdSe/ZnS QDs and MQ, and the CdSe/ZnS QDs were precisely located in the M4 micellar core. The macromolecular aggregates can finally form micelles with a crosslinked structure by the coordination-driven self-assembly process, which is the result of the coordination of multi-sites of MQ and CdSe/ZnS QDs.

**Figure 4:**
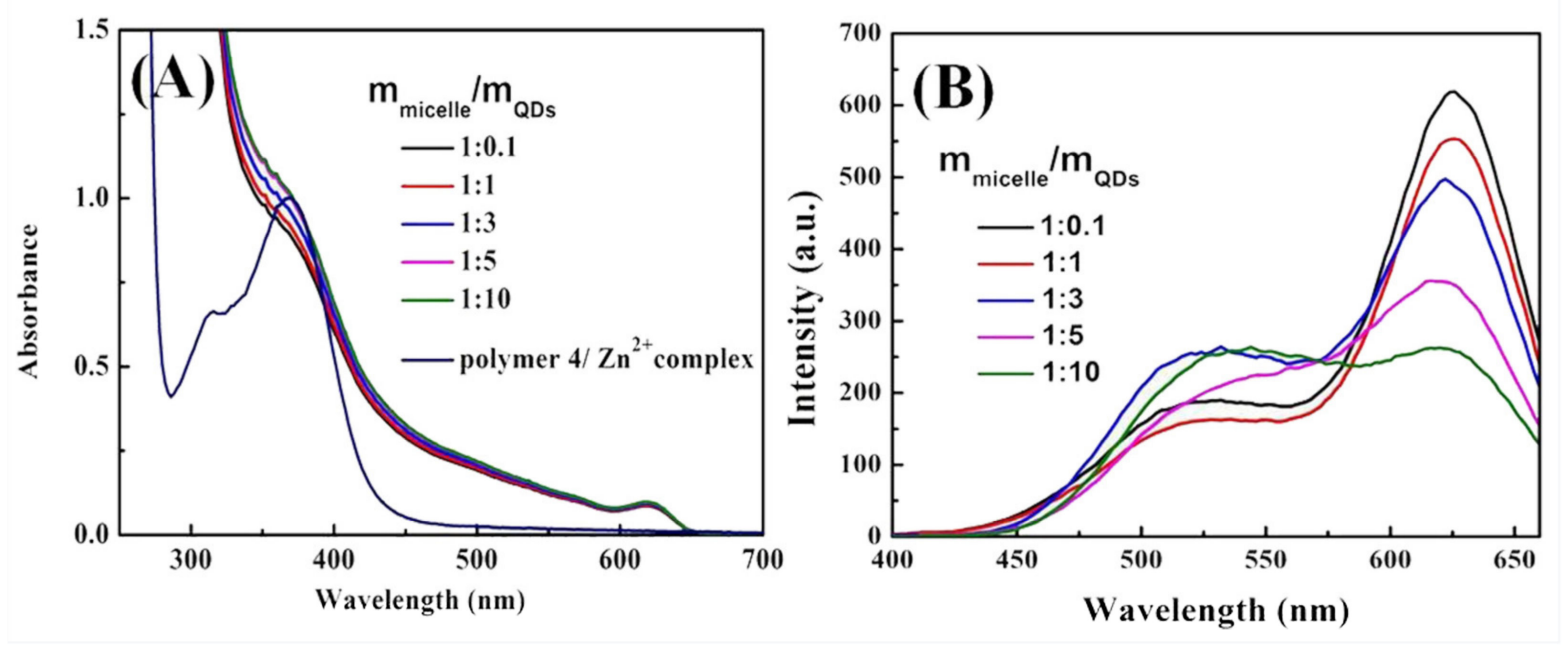
(**A**) UV–Vis absorption spectra and (**B**) PL spectra of CdSe/ZnS@M4-1 with different M4-1/QDs ratio from 1:0.1 to 1:10.

Figure 4A is the UV–Vis absorption spectra of CdSe/ZnS@M4-1 with different M4-1/QDs ratio from 1:0.1 to 1:10. The significant UV–Vis absorption peak at 369 nm was observed (Figure 4A) after the amphiphilic macromolecular ligand P(St_0.8_-co-MQ_0.2_)_61_-b-PNIPAM_15_ (Polymer 4-1) coordinated with Zn^2+^, which derives from the electronic transition between Zn^2+^ and quinoline ring on Polymer 4 [39]. The UV–Vis absorption peak at 369 nm was still observed after Polymer 4-1 and CdSe/ZnS QDs obtained a series of CdSe/ZnS@M4-1 hybrid micelles in different proportions, indicating that the Zn^2+^ on the CdSe/ZnS QDs surface and the quinoline ring in the micelles became stable complexes. This absorption peak at 369 nm is matched with the monocoordinated Zn^2+^-MQ, which indirectly indicates that the MQ in the micelles are complexed to the surface of the QDs in a single coordination form. The absorption peak at 369 nm was significantly enhanced due to more coordination sites that were formed on the CdSe/ZnS QDs surface with the increasing content of the CdSe/ZnS QDs. Figure 4B shows the PL spectra of a series of CdSe/ZnS@M4-1 hybrid micelles in different proportions of Polymer 4-1 and CdSe/ZnS QDs. Since the surface of CdSe/ZnS QDs contains a large amount of Zn^2+^, the MQ motifs on the hydrophobic PSt block of Polymer 4-1 can be coordinated with Zn^2+^ to form the Zn^2+^-MQ complexes on the CdSe/ZnS QDs surface, which in turn produced an effective green light emission layer. Therefore, the CdSe/ZnS@M4-1 hybrid micelles have been successfully built by the coordination-driven self-assembly process that shows dual fluorescence emission characteristics of the intrinsic emission (630 nm) of CdSe/ZnS QDs and the coordination emission (530 nm) of Zn^2+^-MQ complexes on the CdSe/ZnS QDs surface. The fluorescence intensity of CdSe/ZnS QDs was gradually decreased as the increasing content of CdSe/ZnS QDs (Figure 4A), which showed that an effective electron transfer channel was formed between a large number of MQ and CdSe/ZnS QDs in the coordination micelle M4 core. The electron transfer caused the photo-generated electrons of CdSe/ZnS QDs to be trapped by the surface ligand, which in turn caused the fluorescence intensity to gradually decrease. Figure 3B shows the HRTEM images of the single micelle in the CdSe/ZnS@M4-1 hybrid micelles with the ratio of 1:10. The CdSe/ZnS QDs are aggregated in the micelle core due to the induction of MQ motifs, which may also be the cause of the decrease in the intrinsic emission intensity of CdSe/ZnS QDs. However, the change in the coordination emission intensity of the CdSe/ZnS QDs surface is irregular. However, overall, the coordination emission intensity of the CdSe/ZnS QDs surface is increased (except 1:1), which may be due to the increase of luminescent points caused by the increase of coordination sites on the micellar shell with the increase of CdSe/ZnS QDs.

#### 3.4.2. M6@CdTe/ZnS

**Figure 5:**
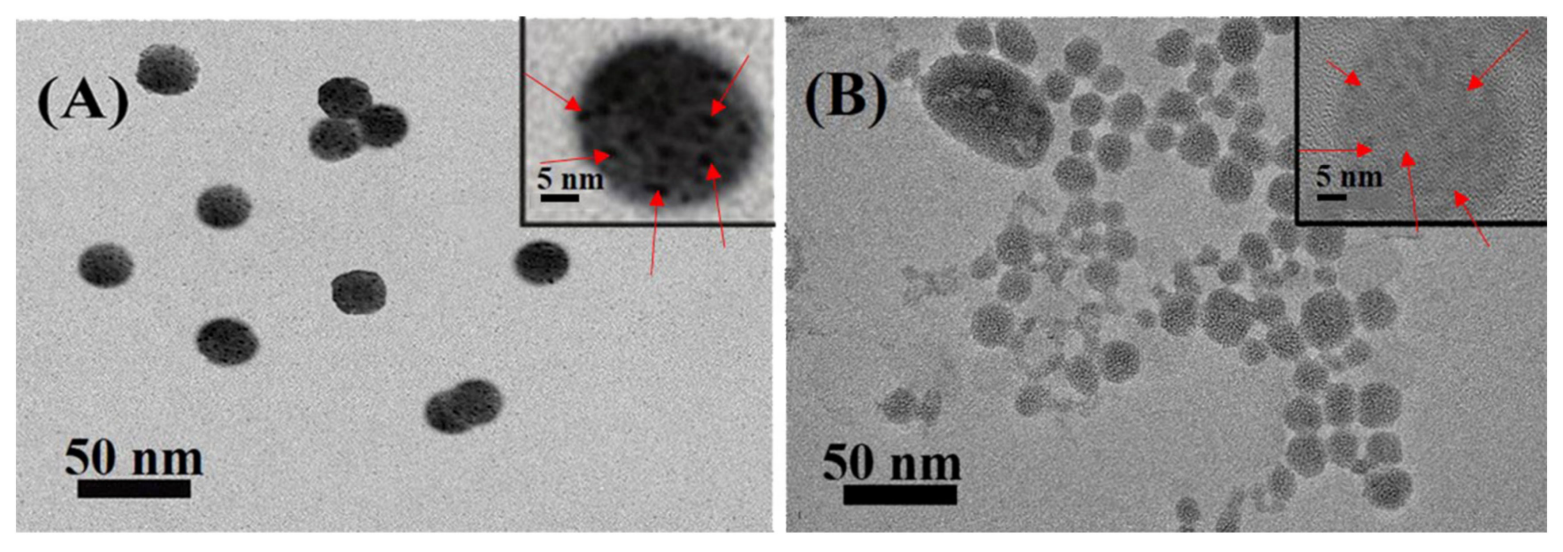
TEM images of M6@CdTe/ZnS with different M6-3/QDs ratio: 1:10 (**A**) and 1:50 (**B**). (Insert: HRTEM of single M6@CdTe/ZnS, the scale bar refers to 5 nm).

The coordination motifs MQ of Polymer 6 (P(NIPAM_0.86_-co-MQ_0.14_)_63_-b-PS_65_) were located in the hydrophilic PNIPAM blocks. Thus, the MQ were located in the shell of the micelles when Polymer 6 self-assembles to form micelles in its selective solvent. Micelles M6-3 were selected as coordination micelles, and water-soluble CdTe/ZnS QDs were added thereto. The M6@CdTe/ZnS hybrid micelles were formed through the coordination-driven interaction between the MQ on the surface of the M6-3 micelles and the Zn^2+^ on the CdTe/ZnS QDs surface. Figure 5A shows the TEM images of M6@CdTe/ZnS with M6-3/QDs ratio of 1:10. When the CdTe/ZnS QDs are introduced into the M6 micelles (1:50), the CdTe/ZnS QDs are uniformly distributed on the micelles surface and particle size is reduced (about 25 nm) compared to the pure M6 micelles size (30 nm). This may be because CdTe/ZnS QDs coordinated with MQ on the surface of the micelles, causing the hydrophilic shell of the micelles to shrink slightly. The CdTe/ZnS QDs are also evenly distributed on the micelle shell with the average particle size of 32 nm (Figure 5B) when more CdTe/ZnS QDs are introduced. It can be observed from the HRTEM images of a single micelle inserted in Figure 5B that M6@/CdTe/ZnS hybrid micelles have been successfully formed by the coordination-driven self-assembly process.

**Figure 6:**
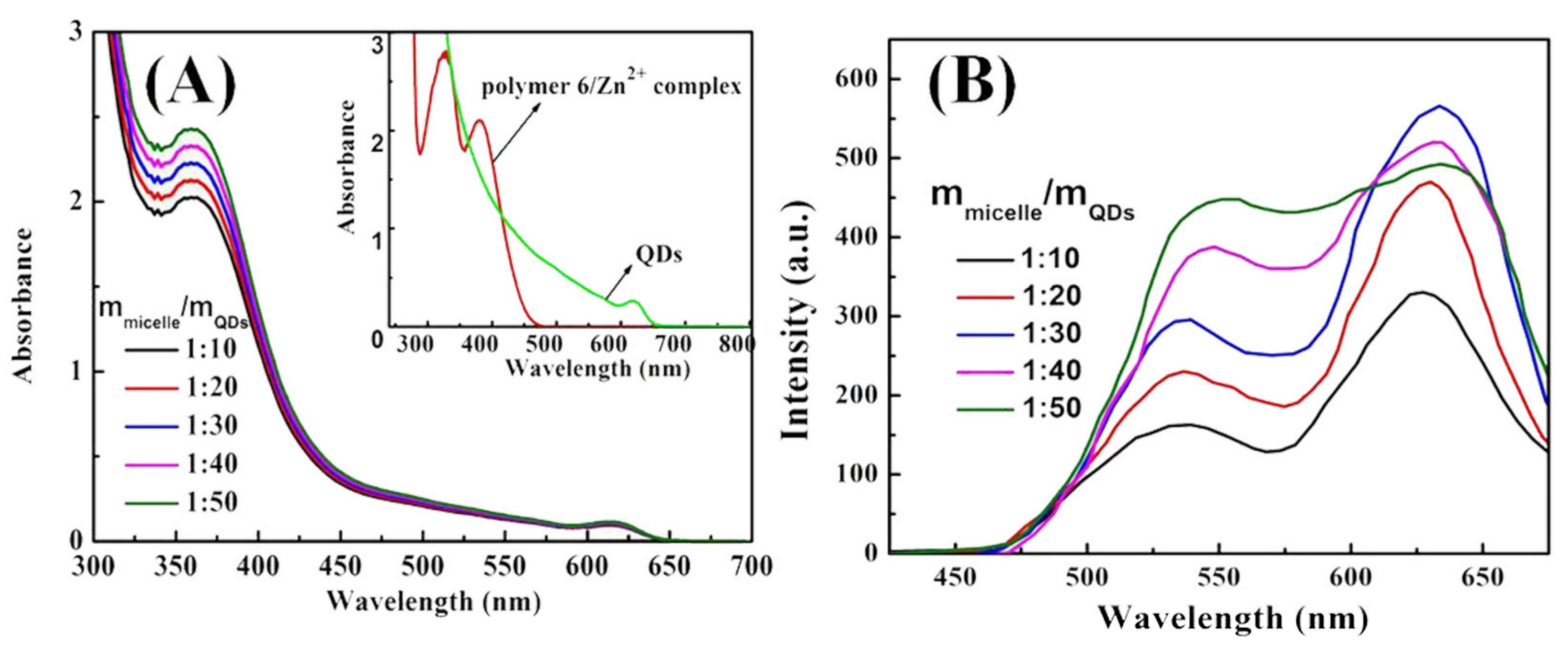
(**A)** UV–Vis absorption spectra and (**B**) PL spectra of M6@CdTe/ZnS with different M6-3/QDs ratio from 1:10 to 1:50.

As shown in Figure 6A, two obviously UV–Vis absorption bands at 360 and 610 nm are observed after the series of M6@CdTe/ZnS hybrids micelles were obtained in different proportions of Polymer 6-3 and CdTe/ZnS QDs. The absorption band at 610 nm is the intrinsic absorption peak of CdTe/ZnS QDs, while the absorption peak at 360 nm is consistent with the UV–Vis absorption peak of the Zn^2+^-MQ complexes, which indicates that the absorption peak at 360 nm in the M6@CdTe/ZnS hybrid micelles originated from the electron transition of Zn^2+^-MQ complexes. In addition, the absorption peak at 360 nm increased with the increasing content of CdTe/ZnS QDs. Figure 6B shows the PL spectrum of a series of M6@CdTe/ZnS hybrid micelles obtained from a different ratio of Polymer 6-3 and CdTe/ZnS QDs. Similarly, the stable green light emission Zn^2+^-MQ complexes were formed by the coordination-driven interaction of the MQ on the micellar shell and the CdTe/ZnS, which in turn produced the successfully built dual-emission M6@CdTe/ZnS hybrid micelles, containing the coordination emission (520 nm) of CdTe/ZnS QDs surface and the intrinsic emission (610 nm) of CdTe/ZnS QDs. The emission intensity of both CdTe/ZnS QDs and surface coordination showed an upward trend with the increasing content of CdTe/ZnS QDs, which was the result of the intrinsic emission of CdTe/ZnS QDs that was enhanced with the increasing content of CdTe/ZnS QDs and the coordination emission intensity was also increased with the increasing MQ-Zn^2+^ coordination sites on the micelles. This is different from the fluorescence emission regularity of CdSe/ZnS@M4-1 hybrid micelles. In CdSe/ZnS@M4-1 hybrid micelles, CdSe/ZnS QDs were located in the core of the micelle due to the coordination-driven effect, which inevitably caused serious aggregation and quenching of CdSe/ZnS QDs. However, compared with CdSe/ZnS@M4-1 hybrid micelles, there was no serious aggregation that caused quenching when CdTe/ZnS QDs were located at the micellar shell by the coordination-driven effect (see Figure 5A) since M6 micelles in M6@CdTe/ZnS hybrid micelles have a large specific surface area. Therefore, the intrinsic emission intensity of the CdTe/ZnS QDs did not appear to be the phenomenon of aggregation quenching. Moreover, the PL intensity of CdTe/ZnS QDs was enhanced when the mass ratio of micelles to CdTe/ZnS QDs was 1:10 to 1:30. The intrinsic luminescence intensity of QDs showed a downward trend when the mass ratio of micelles to QDs was 1:40 and 1:50. It was indicated that there was still a photo-generated electron transfer and electron-hole nonradiative recombination between QDs and surface ligands.

### 3.5. White Light Emission Micelles

**Figure 7:**
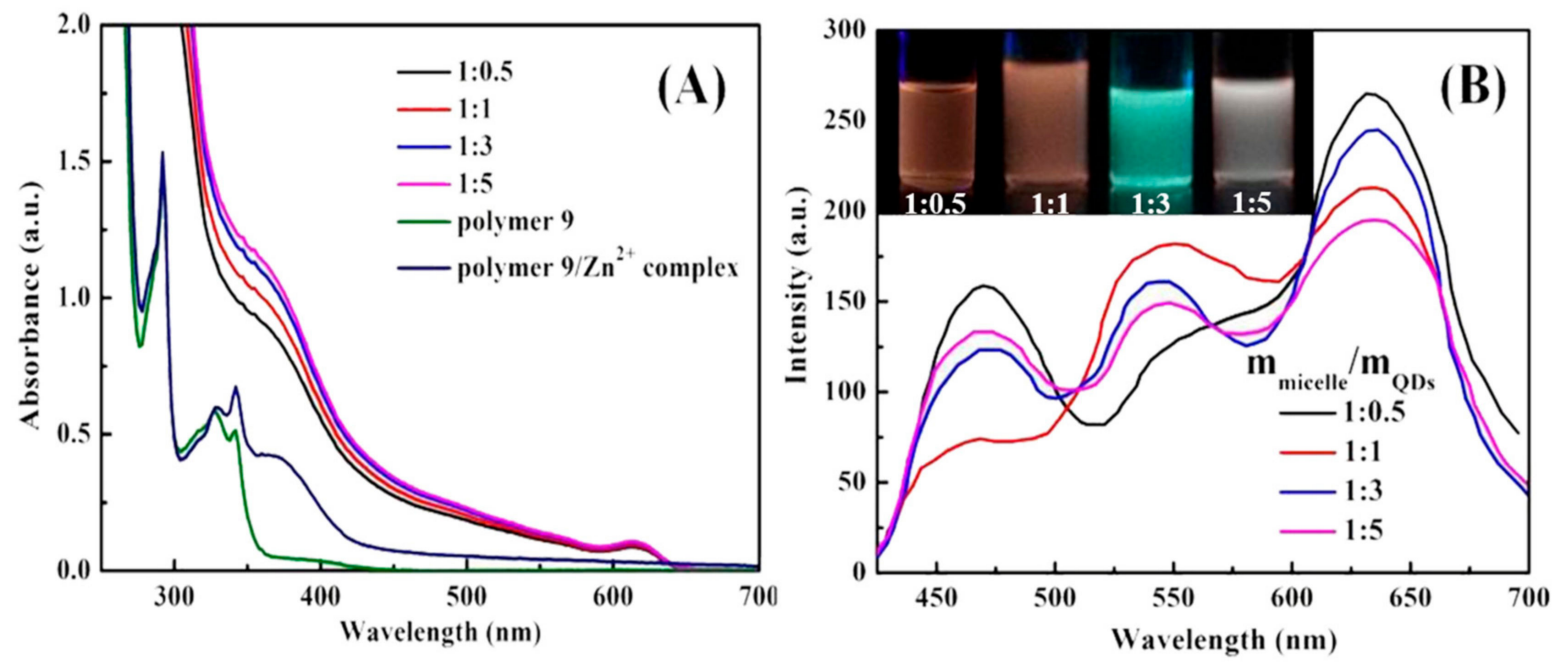
(**A**) UV–Vis absorption spectra of Polymer 9, Polymer 9/Zn^2+^ complex, and CdSe/ZnS@M9 and (**B**) PL spectra of CdSe/ZnS@M9 hybrid micelles with different micelles/QDs ratio from 1:0.5 to 1:5 (Inset: Digital images of CdSe/ZnS@M9 with micelles/QDs ratio from 1:0.5 to 1:5).

The white light emission material is generally formed by superposing three primary colors (red, green, and blue light) in an appropriate ratio, and can also be obtained by complementing two colors, such as blue and orange. Although some studies have obtained white light emission nanoparticles through various means, there are few studies on white emission micelles. Based on the principle of ternary color, the white light emission micelles were obtained in a unique way, that was, white light emission was realized by the synergistic effect of multi-emission in the same micelle. For example, blue emission units (vinyl carbazole, PVK), coordination motifs (MQ), and thermosensitive units (NIPAM) were introduced into the polymer to construct the multifunctional amphiphilic macromolecule ligand P(MQ-co-St)-b-P(NIPAM-PVK) (Polymer 9). Thus, the CdSe/ZnS QDs capped with oleic acid were located in the micellar core through the coordination-driven self-assembly process. Figures S19 and S20 are the ^1^H NMR spectra of the P(MQ-co-NIPAM)-b-(PS-co-PVK) and P(MQ-co-St)-b-(NIPAM-co-PVK), respectively. The chemical shift of 2 at 4.0 ppm is attributed to the hydrogen on the methylidyne of the NIPAM units. The board chemical shifts of 1, 2, b, d, and e at 7.5–8.0 ppm corresponded to the protons of benzene ring on PSt units, the protons PVK units, and the protons on the MQ motifs. The chemical shifts of a, c at 8.5–8.9 ppm are assigned to the protons of pyridine ring on the MQ motifs. The above analysis indicated that the P(MQ-co-St)-b-P(NIPAM-PVK) (Polymer 9) has been successfully synthesized. Figure 7A is the UV–Vis absorption spectra of P(MQ-co-St)-b-P(NIPAM-PVK) (Polymer 9). The significant absorption peak at 291 nm can be observed, which is caused by the π–π* electronic transitions of the pyridine ring on the MQ motifs [38]. In addition, the absorption peak at 326 and 342 nm are attributed to the π–π* electronic transition of the carbazole ring on the PVK units [39]. A new absorption peak at 370 nm except the characteristic absorption peak of the CdSe/ZnS QDs at 610 nm appeared when CdSe/ZnS QDs and Polymer 9 self-assembles in a selective solvent, which originates from the electronic transition between the metal and quinoline rings [38]. This suggested that the monocoordinated Zn^2+^-MQ complexes on the surface of CdSe/ZnS QDs have successfully formed. Moreover, the absorption intensity at 367 nm was enhanced with the increasing content of CdSe/ZnS QDs, and more complexation sites were formed on the surface of CdSe/ZnS QDs.

**Figure 8:**
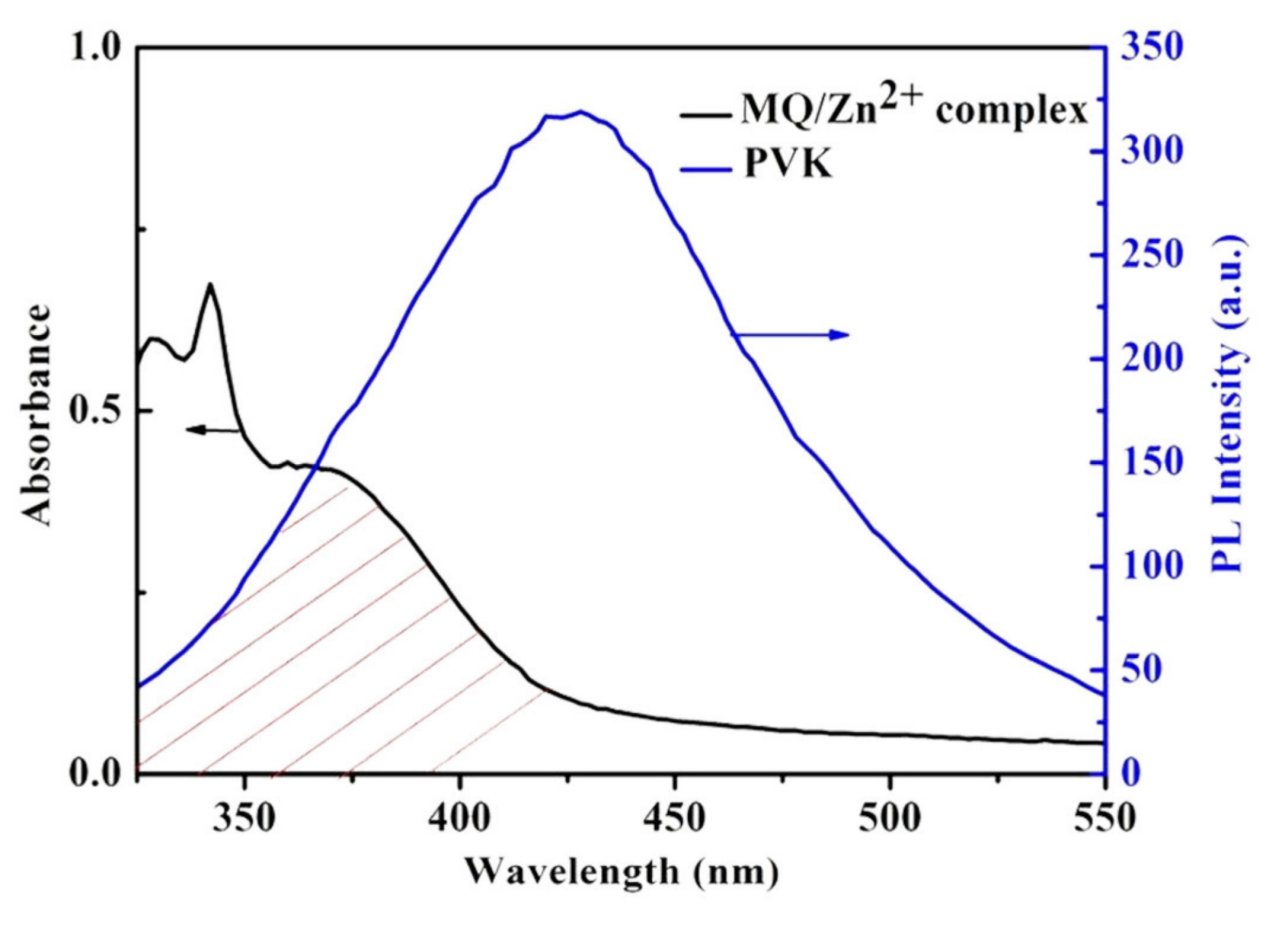
Spectral overlap of absorption (**black line**) of MQ/Zn^2+^ complex and emission spectrum (**blue line**) of PVK.

**Figure 9:**
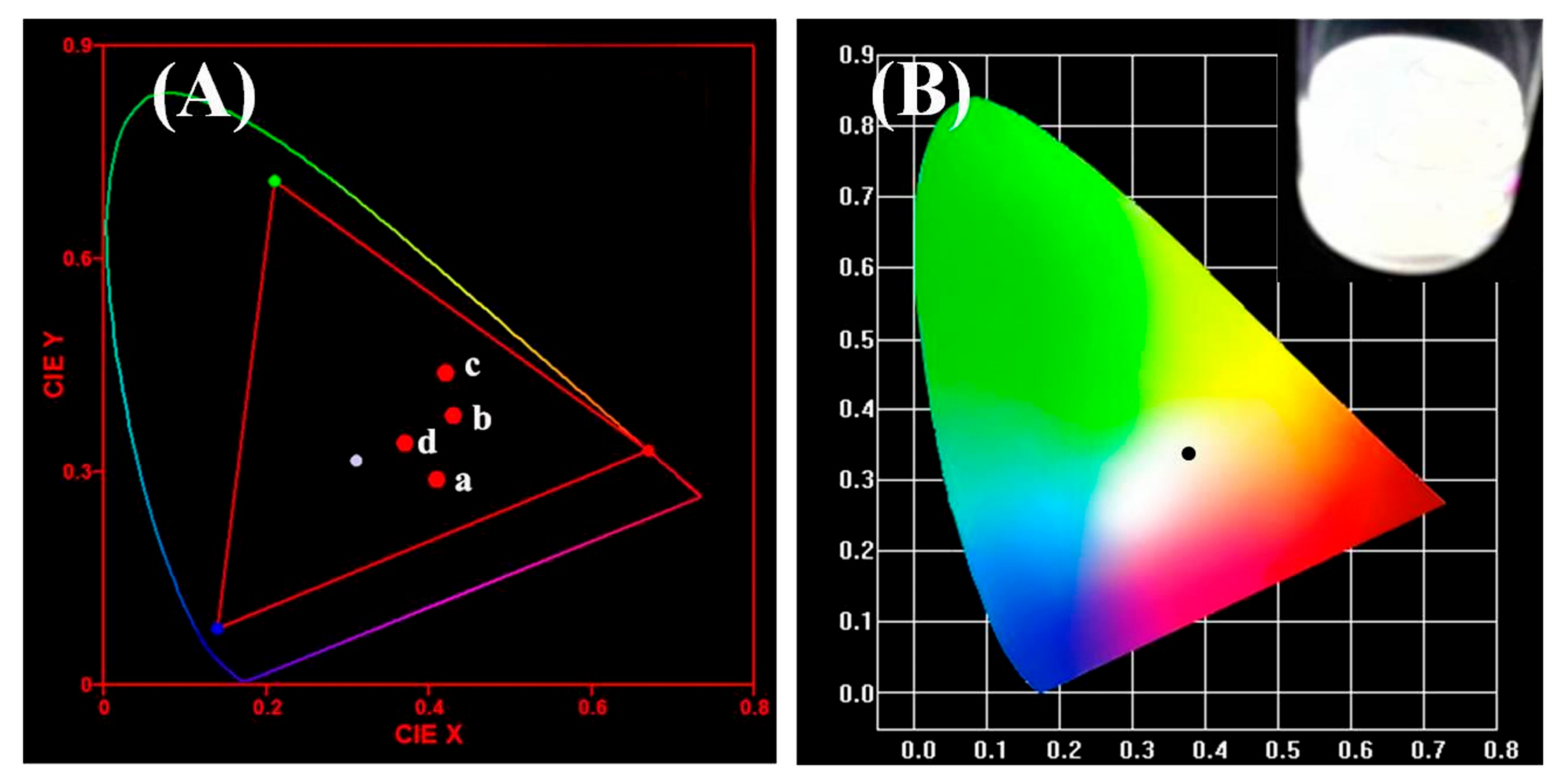
(**A**) CIE chromaticity diagram for CdSe/ZnS@P(MQ-co-St)-b-P(NIPAM-PVK) hybrid micelles with different micelles/QDs ratio (a = 1:0.5, b = 1:1, c = 1:3, d = 1:5) and (**B**) near-white light emission CIE with micelles/QDs ratio of 1:5.

By adjusting the ratio of macromolecular ligands to CdSe/ZnS QDs, the purpose of adjusting the ratio of the three primary colors in CdSe/ZnS@M9 can be achieved. As shown in Figure 7B, when the ratio of macromolecular ligand to CdSe/ZnS QDs is 1:0.5, the emission at 470 nm of PVK units, the emission at 630 nm of CdSe/ZnS QDs motifs, and the coordination emission at about 540 nm of Zn^2+^-MQ complexes were observed, respectively. The surface coordination emission at 540 nm of Zn^2+^-MQ complexes also proved that the macromolecular ligand and CdSe/ZnS QDs were combined by the coordination-driven effect. The fluorescence intensity at 630 nm of CdSe/ZnS QDs was decreased with the increasing content of CdSe/ZnS QDs when the content of macromolecular ligand was controlled to be constant, which may be the result of the electron transfer between macromolecular ligands and CdSe/ZnS QDs. In addition, the surface coordination emission at 540 nm was enhanced with the increasing content of CdSe/ZnS QDs, which was related to the increase of CdSe/ZnS QDs to form more surface Zn^2+^-MQ complexes. Compared with the micelles with a ratio of macromolecular ligands to QDs of 1:0.5, the introduction of more QDs results in the apparent quenching of PVK units fluorescence. This may be caused by energy transfer of the PVK units and the CdSe/ZnS QDs surface ligand. Since fluorescence resonance energy transfer (FRET) is a long-range interaction with respect to the electron transfer process, and may both occur when the distance between the energy acceptor and the donor is less than 10 nm [40]. Figure 8 is the UV–Vis absorption spectrum of Zn^2+^-MQ complexes and the PL emission spectrum of PVK. A large overlap was observed, which satisfied the conditions of the energy transfer of the PVK units to the surface complexes of QDs. Figure 9 is the International Commission on illumination (CIE) 1931 chromaticity coordinate diagram of the above P(MQ-co-St)-b-P(NIPAM-PVK)/QDs hybrid micelles. The chromaticity coordinates of P(MQ-co-St)-b-P(NIPAM-PVK)/QDs hybrid micelles with a mass ratio of 1:0.5, 1:1, 1:3, 1:5 were (0.41, 0.29), (0.43, 0.38), (0.42, 0.44), and (0.37, 0.34), respectively. It is noticed that the CIE (0.37, 0.34) of P(MQ-co-St)-b-P(NIPAM-PVK)/QDs hybrid micelles with a mass ratio of 1:5 is extremely approached to the pure white light (0.33, 0.33) specified by the International Commission on Illumination (CIE) regulations (Figure 9B). According to the principle of white light emission, the near-white light emission of the hybrid micelle originated from the intrinsic red light emission of the CdSe/ZnS QDs, the green light emission of the Zn^2+^-MQ complexes, and the blue light emission of the PVK units on the macromolecular ligands. Due to the synergistic effect of the three light-emitting units, the micelles with the white light emission have been successfully constructed.

**Figure 10:**
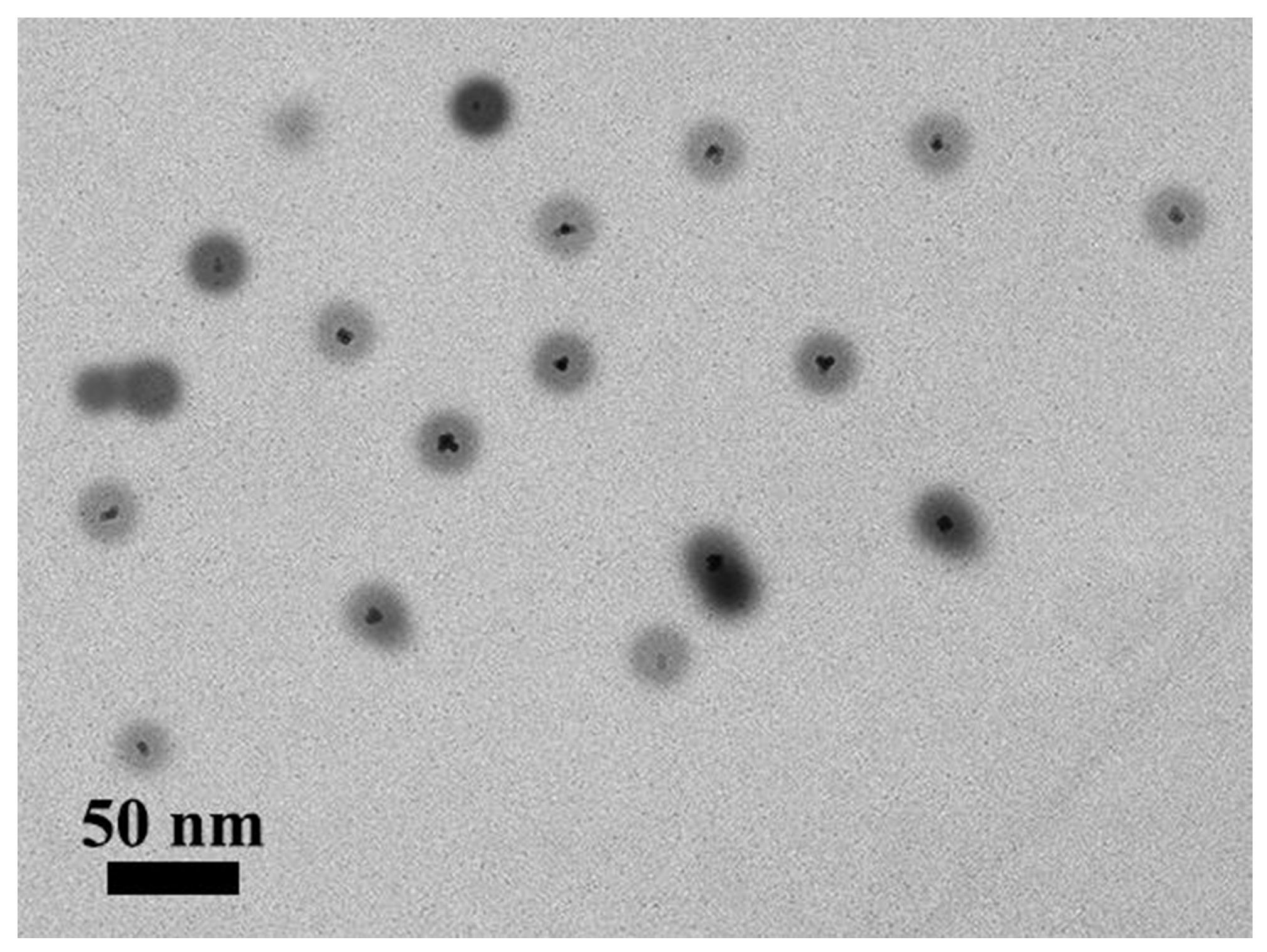
TEM images of CdSe/ZnS@P(MQ-co-St)-b-P(NIPAM-PVK) hybrid micelles.

Figure 10 illustrates the TEM images of CdSe/ZnS@P(MQ-co-St)-b-P(NIPAM-PVK) hybrid micelles at a ratio of 1:5. Due to the coordination motifs MQ that were dispersed in the hydrophobic PSt blocks, the CdSe/ZnS QDs and the macromolecular ligands were self-assembled in a selective solvent to form the hybrid micelles with CdSe/ZnS QDs in the core. The formed hybrid micelles have relatively uniform particle diameters with about 20 nm, and several CdSe/ZnS QDs are distributed in each micelle. This coordination-driven self-assembled hybrid micelles process is stabled through its crosslinked structure, which is the result of the multi-site crosslinking of MQ motifs and CdSe/ZnS QDs in the core internal.

## 4. Conclusions

In summary, we prepared three multifunctional amphiphilic block structure macromolecules ligands by RAFT polymerization using 8-hydroxyquinoline derivatives (MQ) with photoelectric function, isopropylacrylamide (NIPAM) with thermosensitivity, and styrene (St) as monomers. The coordination micelles M4 and M6 with different hydrophilic and hydrophobic blocks ratio were prepared through the self-assembly in the selective solvent water and the corresponding self-assembly morphologies have been studied. The dual-emission hybrid micelles of CdSe/ZnS@M4 and M6@CdTe/ZnS that have been successfully built by the coordination-driven self-assembly process show dual fluorescence emission characteristics of the intrinsic emission of QDs and the coordination emission of Zn^2+^-MQ complexes on the QDs surface. By adjusting the ratio of the macromolecular ligands to QD, the purpose of adjusting the ratio of the three primary colors can be achieved. The white emission micelles which possessed special multi-emission properties in the same micelle were finally constructed through the synergistic effect of the intrinsic red light emission of QDs, the coordination green light of Zn^2+^-MQ complexes, and the blue light emission of PVK. The dual and multi-emission hybrid micelles have great application prospects in ratiometric fluorescent probes and biomarkers.

## Figures and Tables

**Table 1:** Characterization data of copolymers ligand synthesized by the RAFT polymerization.

Samples Copolymer Ligand		M_NMR_ * (KDa)	Mn (KDa)	Mw (KDa)	PDI
—	P(St_49_-co-MQ_12_) Macro-RAFT	8.5	9.2	9.5	1.032
4-1	P(St_0.8_-co-MQ_0.2_)_61_-b-PNIPAM_15_	10.2	9.8	10.5	1.071
4-2	P(St_0.8_-co-MQ_0.2_)_61_-b-PNIPAM_26_	12.6	13.5	16.7	1.237
4-3	P(St_0.8_-co-MQ_0.2_ )_61_-b-PNIPAM_43_	13.4	14.1	18.2	1.290
4-4	P(St_0.8_-co-MQ_0.2_)_61_-b-PNIPAM_51_	14.3	15.3	20.5	1.339
—	P(NIPAM_54_-co-MQ_9_)Macro-RAFT	8.6	9.3	9.5	1.021
6-1	P(NIPAM_0.86_-co-MQ_0.14_)_63_-b-PS_27_	11.5	11.4	12.1	1.061
6-2	P(NIPAM_0.86_-co-MQ_0.14_)_63_-b-PS_32_	11.9	12.3	13.8	1.121
6-3	P(NIPAM_0.86_-co-MQ_0.14_)_63_-b-PS_65_	15.4	15.9	17.3	1.088
6-4	P(NIPAM_0.86_-co-MQ_0.14_)_63_-b-PS_86_	17.6	18.2	19.1	1.049

* M_NMR_ were the molecular weight calculated on the basis of ^1^H NMR results.

## Supplementary Materials

The following are available online at https://www.mdpi.com/1996-1944/13/2/440/s1. Figure S1: TEM images of (a) CdSe, (b) CdSe/ZnS QDs. Figure S2: XRD patterns of the CdSe and CdSe/ZnS QDs. Figure S3: TEM images (a) and HRTEM image (b) of CdTe/ZnS QDs. Figure S4: GPC curves of Polymer 4 and their corresponding macro-RAFT agents. Figure S5: GPC curves of Polymer 6 and their corresponding macro-RAFT agents. Figure S6: FTIR spectra of P(MQ-co-St)-RAFT (3) and P(MQ-co-St)-b-PNIPAM (4). Figure S7: FTIR spectra of P(MQ-co-NIPAM)-RAFT (5) and P(MQ-co-NIPAM)-b-PS(6). Figure S8: ^1^H-NMR spectra of DDMAT in CDCl_3_. Figure S9: ^1^H-NMR spectra of P(MQ-co-St)-RAFT (polymer 3) in CDCl_3_. Figure S10: ^13^C-^1^H COSY spectra of P(MQ-co-St)-RAFT (polymer 3) in CDCl_3_. Figure S11: ^1^H-NMR spectra of P(MQ-co-St)-b-PNIPAM (polymer 4-1) in CDCl_3_. Figure S12: ^1^H-NMR spectra of P(MQ-co-St)-b-PNIPAM (polymer 4-2) in CDCl_3_. Figure S13: ^1^H-NMR spectra of P(MQ-co-St)-b-PNIPAM (polymer 4-3) in CDCl_3_. Figure S14: ^1^H-NMR spectra of P(MQ-co-St)-b-PNIPAM (polymer 4-4) in CDCl_3_. Figure S15: ^1^H-NMR spectra of P(MQ-co-NIPAM)-RAFT (polymer 5) in CDCl_3_. Figure S16: ^1^H-NMR spectra of P(MQ-co-NIPAM)-b-PS (polymer 6-1) in CDCl_3_. Figure S17: ^1^H-NMR spectra of P(MQ-co-NIPAM)-b-PS (polymer 6-2) in CDCl_3_. Figure S18: ^1^H-NMR spectra of P(MQ-co-NIPAM)-b-PS (polymer 6-3) in CDCl_3_.
