# Supplementary material for: Dual and Multi-Emission Hybrid Micelles Realized through Coordination-Driven Self-Assembly
Source: Materials (Basel). 2020 Jan 16;13(2):440. doi: 10.3390/ma13020440 (PMC7014128; doi:10.3390/ma13020440)
Supplement: Supplementary file 1 [file materials-13-00440-s001.pdf]

materials

Supplementary Information

Dual and Multi-Emission Hybrid Micelles Realized through Coordination-Driven Self-Assembly

Youxiong Zheng [1], Yan Tang[1], Jianwei Yu [1], Lan Xie [1], Huiyou Dong [1], Rongsheng Deng [1], Fuhua Jia [1], Bingxin Liu [1,*], Li Gao [1,*] and Junyuan Duan [2,*]

[1] Qinghai Provincial Key Laboratory of New Light Alloys, Qinghai Provincial Engineering Research Center of High Performance Light Metal Alloys and Forming, Qinghai University, Xining 810016, China; youxiongzheng@126.com (Y.Z.); a1172050909@163.com (Y.T.); yjw13897471771@163.com (J.Y.); 18990052263@163.com (L.X.); zblzc10@163.com (H.D.); ydxrdsr@126.com (R.D.); hellofuhua@126.com (F.J.)

[2] State Key Laboratory of Material Processing and Die & Mould Technology, School of Materials Science and Engineering, Huazhong University of Science and Technology, Wuhan 430074, China

* Correspondence:Correspondence: liubx408@nenu.edu.cn (B.L.); 2007990030@qhu.edu.cn (L.G.); junyduan@hust.edu.cn (J.D.); Tel.: +86-186-9723-5043 (B.L.)

Preparation of CdSe/ZnS QDs capped with oleic acid

Preparation of Cd precursor fluid, Se precursor fluid, Zn precursor fluid, and S precursor fluid preparation by reference to previous methods [1].

Preparation of CdSe QDs: 20 mL of Se precursor fluid and 20 ml of ODE were added in a 50 mL three-necked flask, and degassed with an oil pump at 120 °C for 30 min. Turned off the oil pump, and turned on the N_2 ball and heated to 280 °C. 4 mL of Cd precursor fluid was quickly injected into the above reaction solution, and the heating was stopped after reacting at 260 °C for 5 min. When the reaction liquid was cooled to room temperature, a large amount of precipitant which was prepared by a mixture of ethyl acetate and methanol in a ratio of EA: MeOH: QDs=0.75:0.75:1 was poured into the reaction liquid to precipitate. The obtained precipitate was centrifuged and washed by $CHCl_3$ with six times. The red CdSe solution was finally obtained through the precipitate was dispersed in chloroform.

Preparation of CdSe/ZnS QDs: 1 g ODA, 5 mL ODE and CdSe chloroform solution were added in a 50 ml three-necked flask. The mixture was degassed for 1 h with an oil pump at 150 °C to remove chloroform and raised the temperature to 200 °C under N_2 atmosphere. 0.58 mL of Zn precursor solution and S precursor solution were slowly add to the reaction flask and reacted for 10 min. 0.78 mL of Zn precursor solution and S precursor solution were also slowly added dropwise to the reaction flask for 10 min. In addition, 1 mL of Zn precursor solution and S precursor solution were added, and the heating was stopped after the reaction was carried out for 20 min. when the reaction solution was cooled to room temperature, it was added to a large amount of acetone to precipitate, and the obtained precipitate was centrifuged and washed by chloroform or methanol. After repeated five times of this procedure, the core-shell structure CdSe/ZnS QDs were obtained with dried under vacuum. [1,2].

Preparation of water phase CdTe/ZnS QDs

Preparation of Te Precursor (NaHTe): 250 mg Te powder and 2 mL deionized water were added to a 10 mL vial. After the mixture was degassed with argon gas for 15 min, 160 mg of $NaBH_4$ was added to carry out the following reaction:

$$4NaBH_4 + 2Te + 7H_2O \rightarrow 2NaHTe + Na_2B_4O_7 + 14H_2\uparrow$$

Since the reaction was exothermic, it was carried out in an ice bath until the solution becomes grayish (this step was essential for the solution. When it turned purple, it may be oxidized).

Preparation of CdTe QDs [1]: 50 mL 0.01 M $CdCl2·2.5H_2O$ and 50 mL 0.01 M MPA was added to a 500 mL single-necked flask, and deionized water was added to form a 400 mL solution when pH was adjusted to 9 by 0.1 M NaOH. After the mixture was degassed with argon gas for 20 min, 0.25 mL of off-white NaHTe solution was quickly added and magnetic stirred for 10 min, then it was heated to 100 °C to reflux. The CdTe QDs of the desired wavelength were obtained by controlling the reflux time. The obtained QDs stored in the dark after dialyzed for one week.

Preparation of Core-shell structure CdTe/ZnS QDs [2]. 40 mg of CdTe were added to the 50 mL aqueous solution containing 1mmol/L $ZnCl_2$ and 4mmol/L GSH when the pH was adjusted to 8. The above mixture was heated to 100 °C and refluxed for 10 min. This process did not require argon protection. The obtained QDs stored in the dark after dialyzed for one week, and then put aside for later use.

Preparation of RAFT reagent DDMAT

Trithiocarbonate S-1-dodecyl-S'-(α, α'-dimethyl-α"-acetic acid) (DDMAT) was used as the RAFT reagent. Specific reference to the improved method of literature [3]: 8.76 g of dodecyl mercaptan (0.04 mol), 19.24 g of acetone (0.331 mol) and 0.512 g of CTACl (0.0016 mol) were mixed and introduced N_2 at 10 °C. 3.35 g of NaOH solution (50%, 0.042 mol) was slowly added dropwise to the above mixed solution to give a white milky appearance. After stirring for 15 min, 3.04 g of CS_2 (0.04 mol) and 4.04 g of acetone (0.069 mol) were added and the emulsion changed from yellow to orange. 7.13 g of chloroform (0.6 mol) was added to the emulsion after 10 min, and 16 g of NaOH solution (50%, 0.2 mol) was also slowly added dropwise over 30 min. The resulting emulsion was red after overnight. 60 mL of water was added the above emulsion, acidified with 10 mL of concentrated hydrochloric acid and acetone was taken out through N_2 to obtain solid. The solid was filtered through a Buchner funnel, and then dissolved by adding 100 mL of isopropyl alcohol. The undissolved solid DDMAT was removed by filtration. The product was obtained by concentrated evaporation on a rotary evaporator. The crude product was recrystallized three times with n-hexane to give yellow needle crystals. mp: 64 °C. 1 H NMR (500 MHz, $CDCl_3$, δ, ppm): 0.99 (t, 3H), 1.37 1.47 (m, 20H), 1.75 (s, 6H), 3.42 (t, 2H).

Results and discussion

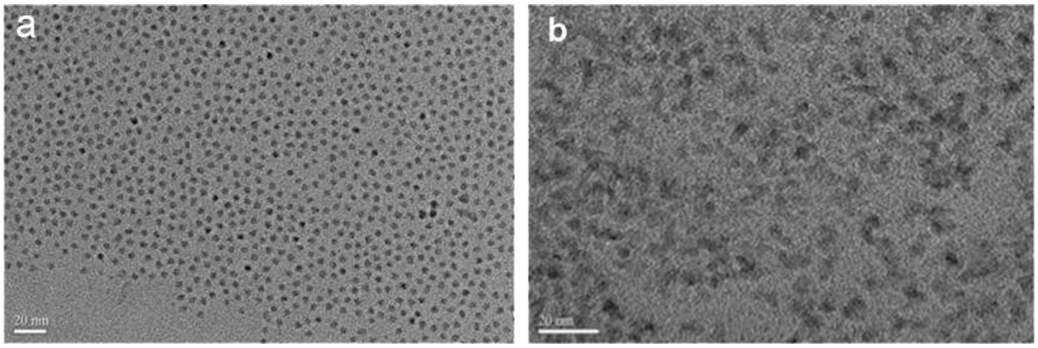

Figure 1. TEM images of (**a**) CdSe, (**b**) CdSe/ZnS QDs.

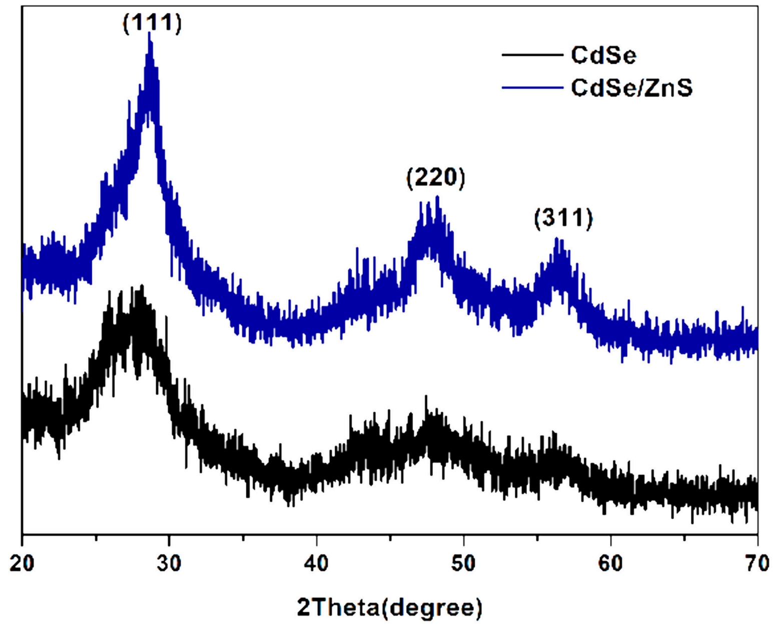

Figure 2. XRD patterns of the CdSe and CdSe/ZnS QDs.

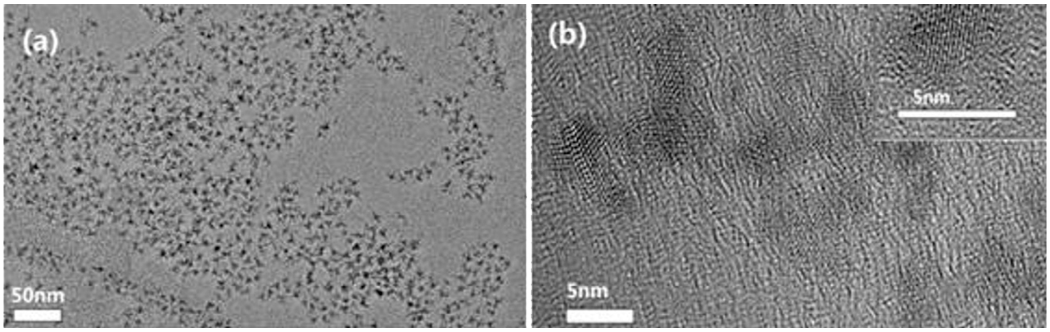

Figure 3. TEM images (**a**) and HRTEM image (**b**) of CdTe/ZnS QDs.

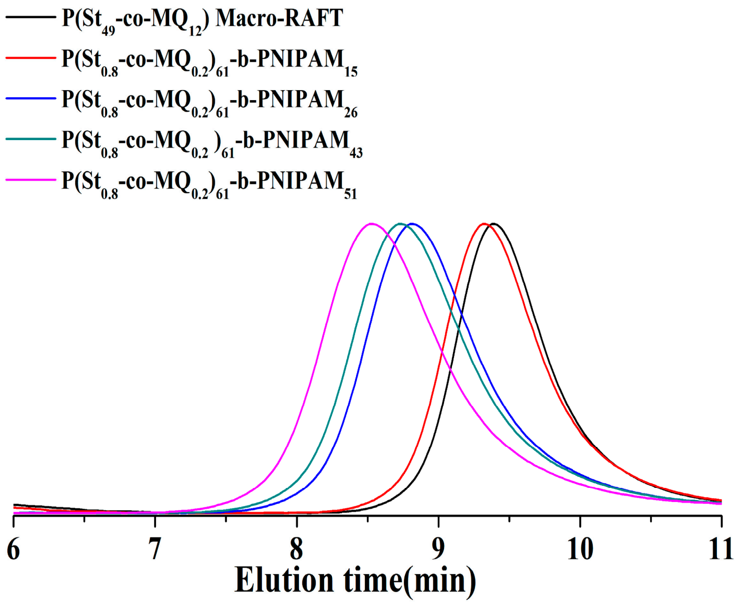

Figure 4. GPC curves of Polymer 4 and their corresponding macro-RAFT agents.

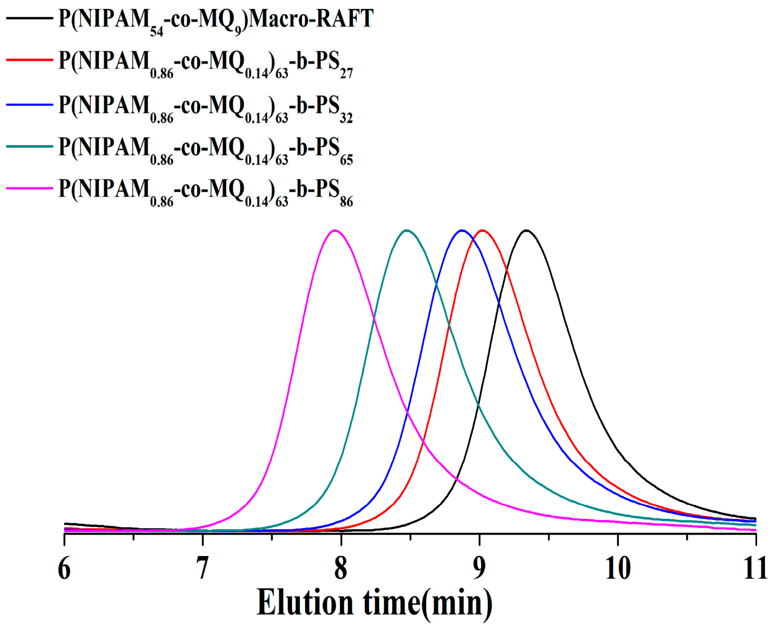

Figure 5. GPC curves of Polymer 6 and their corresponding macro-RAFT agents.

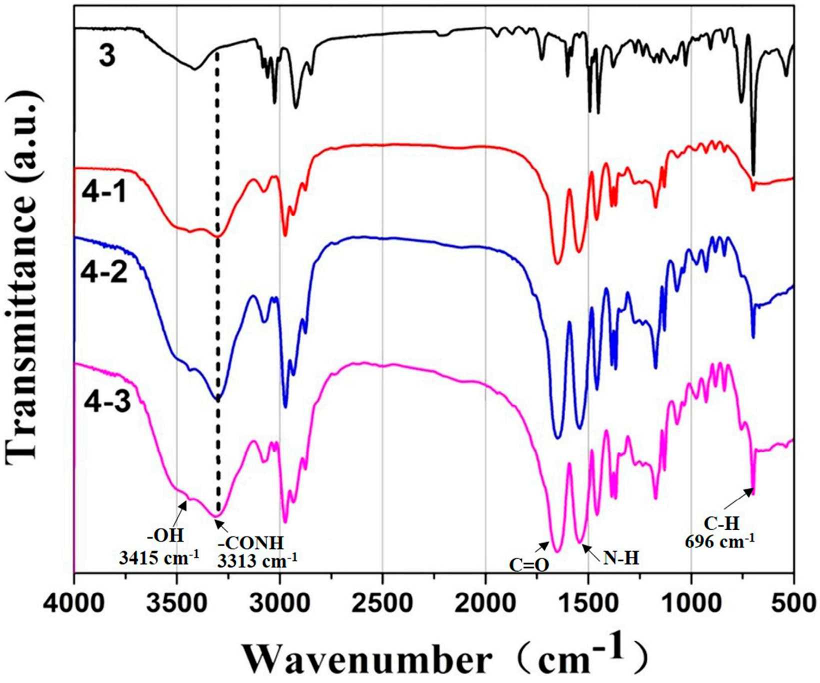

Figure 6. FTIR spectra of P(MQ-co-St)-RAFT (3) and P(MQ-co-St)-b-PNIPAM (4).

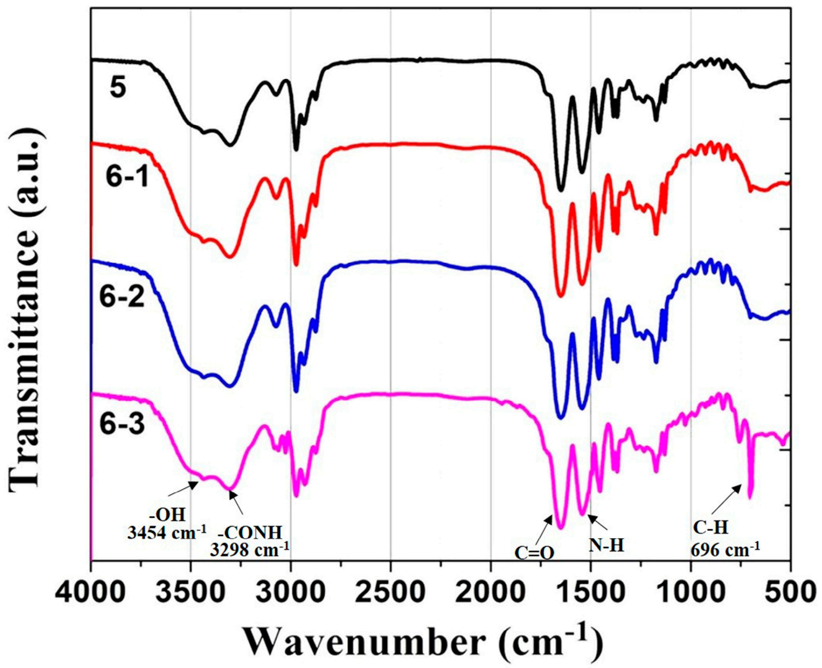

Figure 7. FTIR spectra of P(MQ-co-NIPAM)-RAFT (5) and P(MQ-co-NIPAM)-b-PS(6).

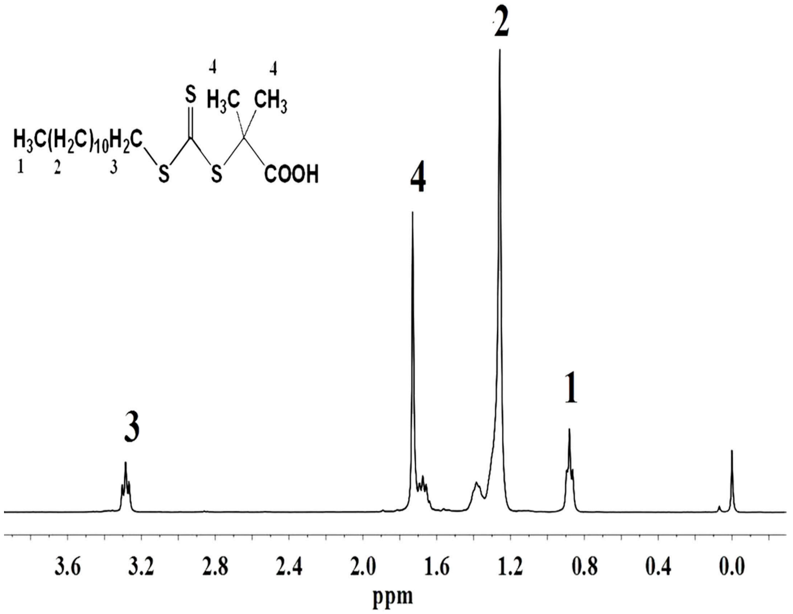

Figure 8. ^{1}H-NMR spectra of DDMAT in $CDCl_3$.

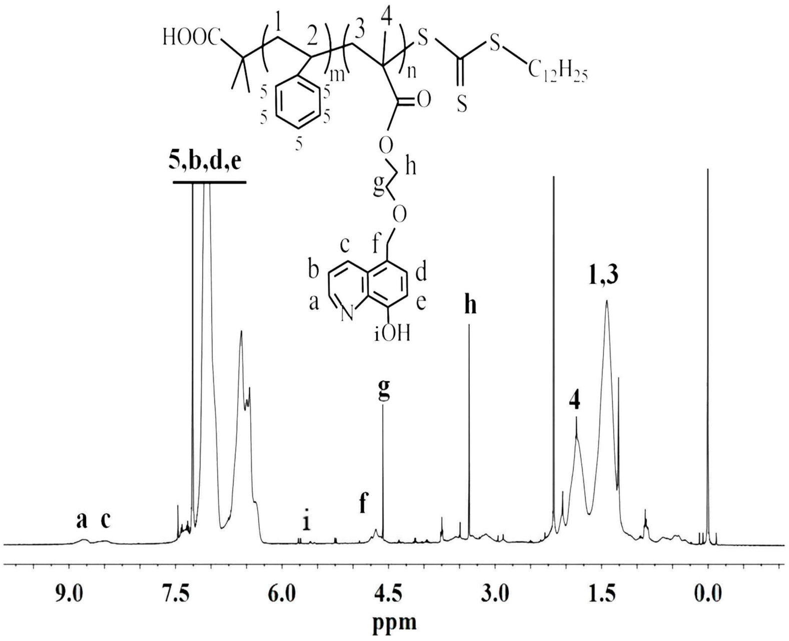

Figure 9. ^{1}H-NMR spectra of P(MQ-co-St)-RAFT (polymer 3) in $CDCl_3$.

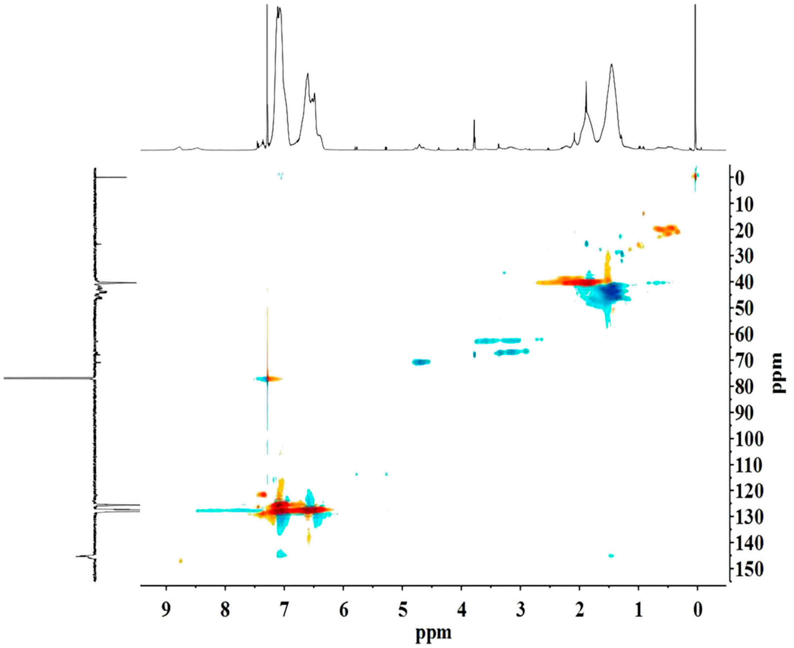

Figure 10. ^{13}C-^{1}H COSY spectra of P(MQ-co-St)-RAFT (polymer 3) in $CDCl_3$.

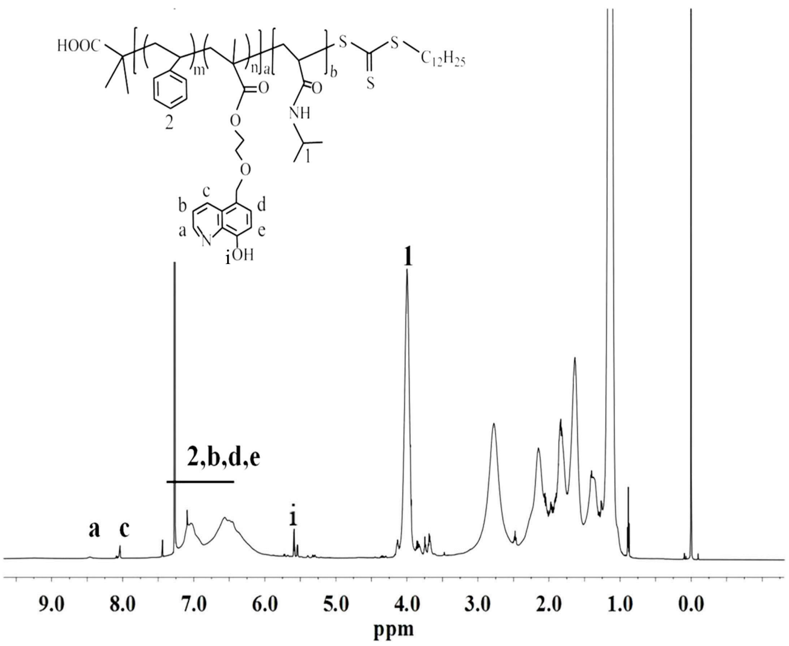

Figure 11. ^{1}H-NMR spectra of P(MQ-co-St)-b-PNIPAM (polymer 4-1) in $CDCl_3$.

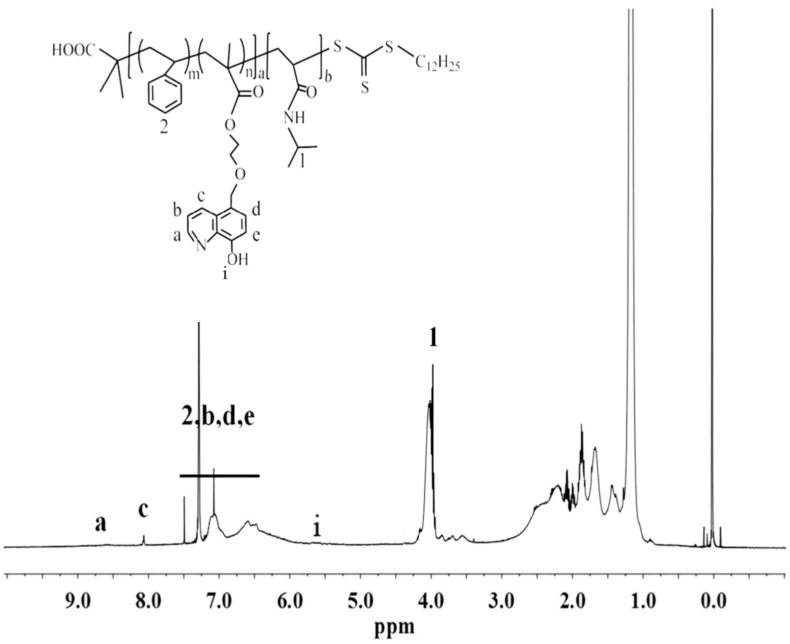

Figure 12. ^{1}H-NMR spectra of P(MQ-co-St)-b-PNIPAM (polymer 4-2) in $CDCl_3$.

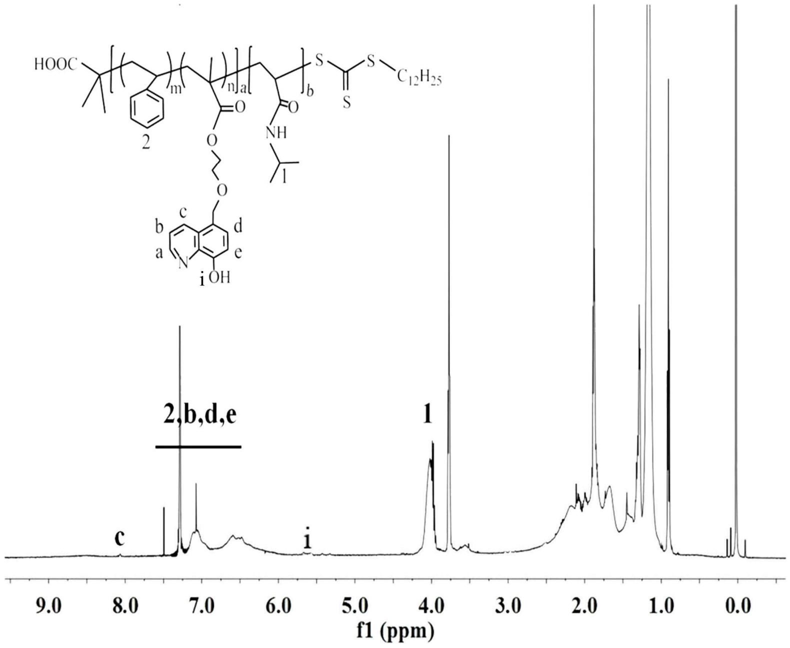

Figure 13. ^{1}H-NMR spectra of P(MQ-co-St)-b-PNIPAM (polymer 4-3) in $CDCl_3$.

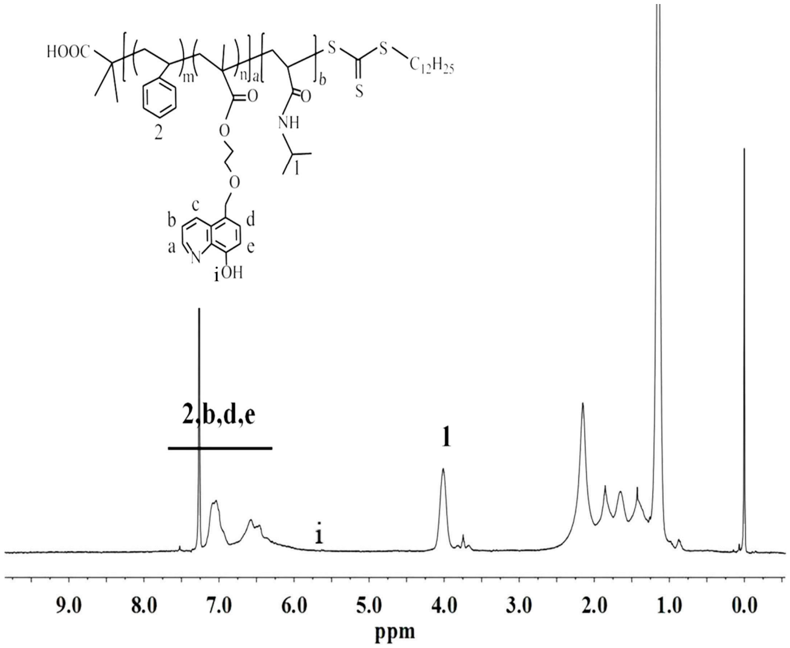

Figure 14. ^{1}H-NMR spectra of P(MQ-co-St)-b-PNIPAM (polymer 4-4) in $CDCl_3$.

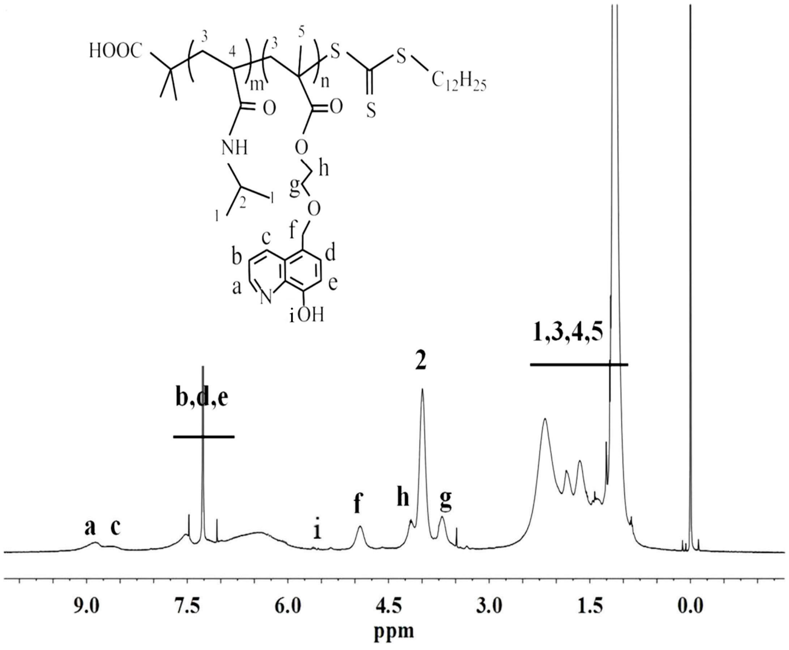

Figure 15. ^{1}H-NMR spectra of P(MQ-co-NIPAM)-RAFT (polymer 5) in $CDCl_3$.

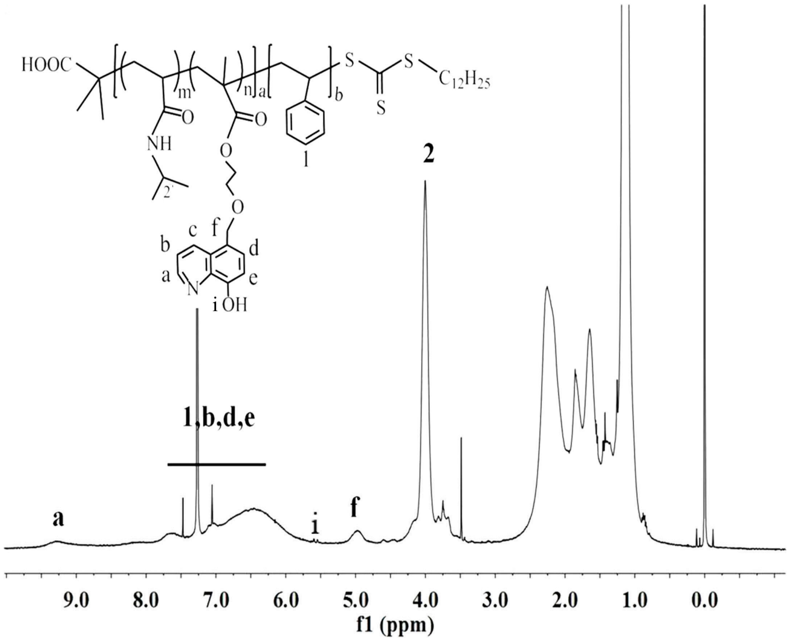

Figure 16. ^{1}H-NMR spectra of P(MQ-co-NIPAM)-b-PS (polymer 6-1) in $CDCl_3$.

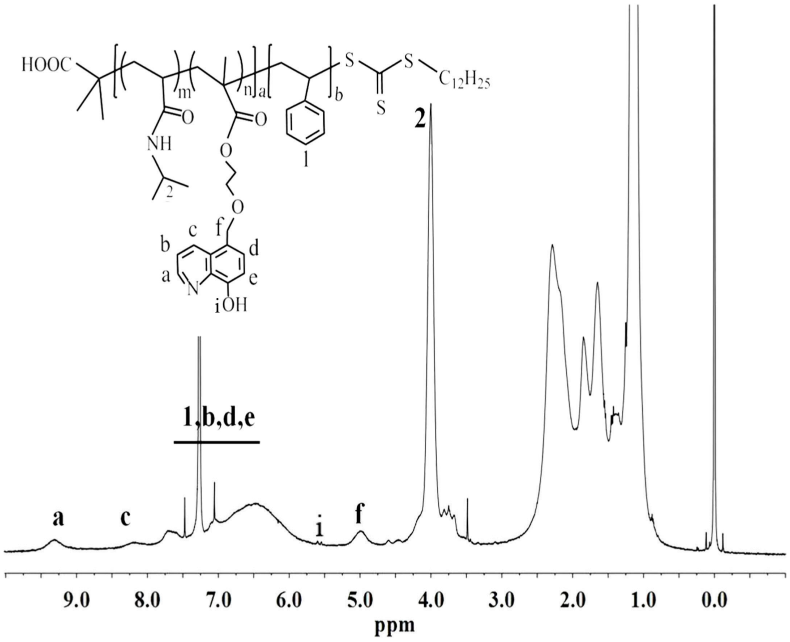

Figure 17. ^{1}H-NMR spectra of P(MQ-co-NIPAM)-b-PS (polymer 6-2) in $CDCl_3$.

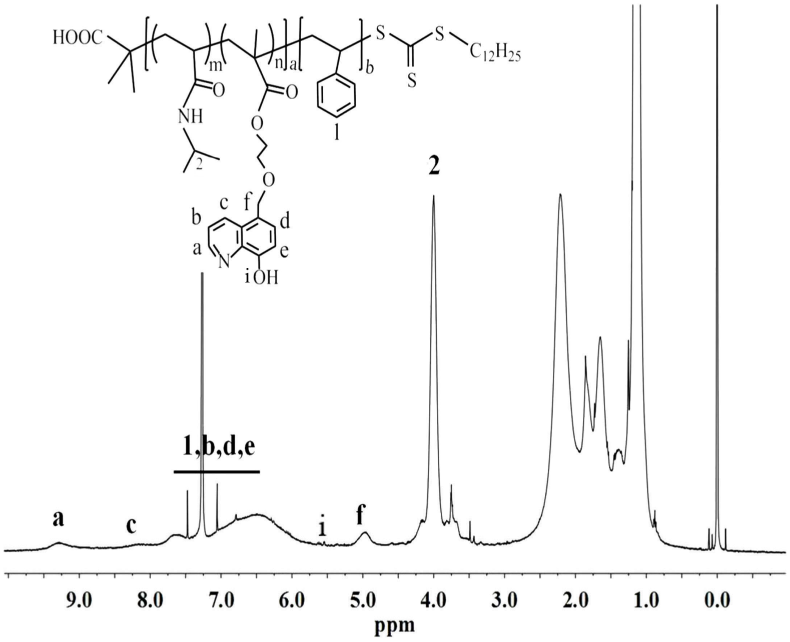

Figure 18. ^{1}H-NMR spectra of P(MQ-co-NIPAM)-b-PS (polymer 6-3) in $CDCl_3$.

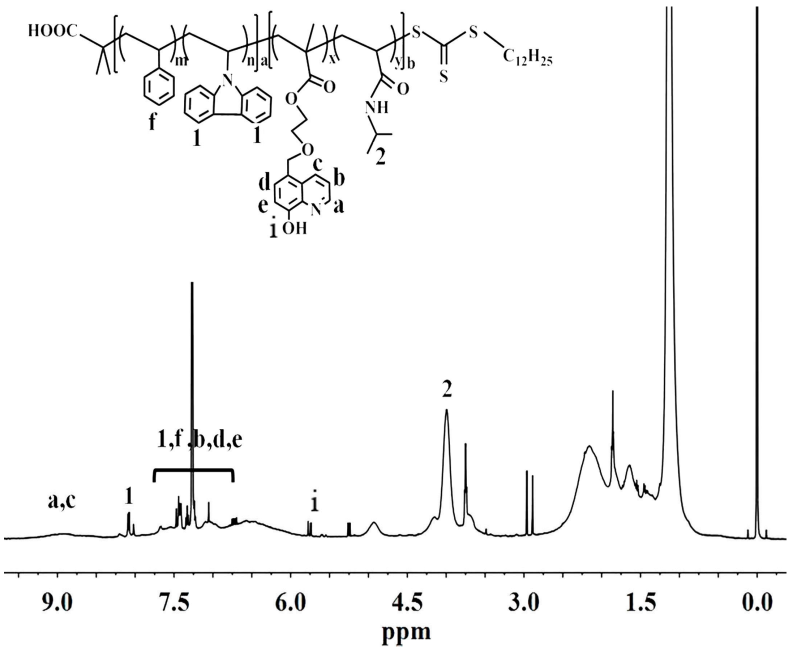

Figure 19. ^{1}H-NMR spectra of P(MQ-co-NIPAM)-b-(PS-co-PVK) (polymer 7) in $CDCl_3$.

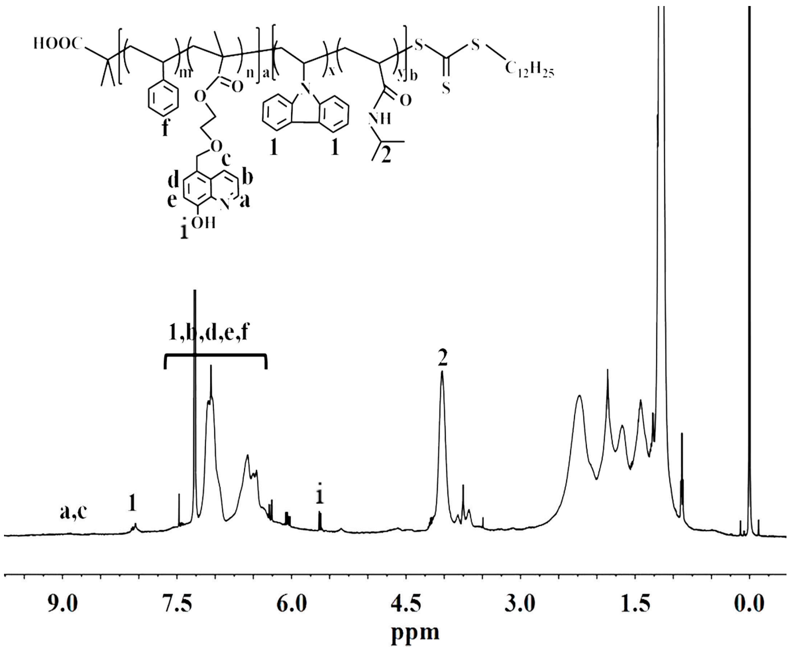

Figure 20. ^{1}H-NMR spectra of P(MQ-co-St)-b-(NIPAM-co-PVK) (polymer 9) in $CDCl_3$.

References

1. Zhang H.; Zhou Z.; Yang B.; Gao M. The Influence of Carboxyl Groups on the Photoluminescence of Mercaptocarboxylic Acid-Stabilized CdTe Nanoparticles. *Journal of Physical Chemistry B*. **2003**, *107*, 8–13.
2. Liu Y.-F.; Yu J.-S. In situ synthesis of highly luminescent glutathione-capped CdTe/ZnS quantum dots with biocompatibility. *Journal of colloid and interface science*. **2010**, *351*, 1–9.
3. Lai J.T.; Filla D.; Shea R. Functional Polymers from Novel Carboxyl-Terminated Trithiocarbonates as Highly Efficient RAFT Agents. *Macromolecules*. **2002**, *35*, 6754–6756.

© 2020 by the authors. Submitted for possible open access publication under the terms and conditions of the Creative Commons Attribution (CC BY) license (http://creativecommons.org/licenses/by/4.0/).
